# Gendered Parenthood-Employment Gaps from Midlife: A Demographic Perspective Across Three Different Welfare Systems

**DOI:** 10.1007/s10680-024-09699-2

**Published:** 2024-05-24

**Authors:** Angelo Lorenti, Jessica Nisén, Letizia Mencarini, Mikko Myrskylä

**Affiliations:** 1 Max Planck Institute for Demographic Research, Rostock, Germany; 2 Max Planck-University of Helsinki Center for Social Inequalities in Population Health, Rostock, Germany; 3 Invest Research Flagship, University of Turku, Turku, Finland; 4 Dondena Centre for Research on Social Dynamics and Public Policy and Department of Social and Political Sciences, Bocconi University, Milan, Italy; 5 Department of Social Research, University of Helsinki, Helsinki, Finland

**Keywords:** Employment gaps by parity, Gender employment expectancies, Parenthood employment gaps, Midlife employment trajectories, Multistate life table, Cross country comparison

## Abstract

Women’s labor force participation has increased in Western countries, but gender gaps remain, especially among parents. Using a novel comparative perspective, we assess women’s and men’s employment trajectories from midlife onward by parity and education. We provide insights into the gendered parenthood-employment gaps examining the long-term implications of parenthood beyond the core childbearing ages by decomposing years lived between ages 40–74, in years of employment, joblessness, and retirement. Using multistate incidence-based life tables, we compare different cultural and institutional contexts: Finland, Italy, and the USA. Our results document large cross-national variation, with education playing a key role. In Finland, the number of years of employment increases with parity for women and men, and the gender gap is small; in the USA, the relationship between parity and years of employment is relatively flat, although a gender gap emerges among those with two or more children; in Italy, the number of years of employment decreases sharply for women as parity increases, while it increases for men. Notably, education has a similar positive impact on years of employment across all groups in Finland. In contrast, in the USA and Italy, the gender gap is only half as large among highly educated mothers as it is among low educated mothers. The employment trajectories of childless women and men differ greatly across countries.

**Supplementary Information:**

The online version contains supplementary material available at 10.1007/s10680-024-09699-2.

## Introduction

Despite the remarkable growth in women’s labor force participation over the last century (e.g., Goldin, 2014), combining the roles of mother and worker continues to be difficult, as indicated by large employment gaps between mothers and fathers of children under age 18 in USA (21 percentage points, Bureau of Labor Statistics, 2022) and in EU countries (18 percentage points, Eurostat, 2022).

There is a vast body of research documenting the motherhood wage and occupational penalties. Several studies have shown that compared to fathers and childless women, mothers often experience more discontinuous careers and encounter greater challenges in career progression. The negative effect of motherhood on employment and wages operates through different mechanisms, with the most immediate being the short-term interruption in paid work following childbirth (Gangl & Ziefle, 2009) and the loss of work experience, which can have lasting consequences over the long term (Kahn et al., 2014). Working fewer hours or leaving work to care for children can endanger women’s future salaries and career prospects (Killewald & Zhuo, 2019; Sigle-Rushton & Waldfogel, 2007), increasing their risk of economic insecurity at older ages (Muller et al., 2020). The consequences of mothers leaving employment or reducing their levels of labor market participation are becoming increasingly critical for Western countries, where population aging is leading to calls for a substantial extension of working life to ensure the sustainability of welfare systems.

Still, there is limited research on how mothers’ employment trajectories unfold from midlife until retirement (Muller et al., 2020), and even more limited is the research examining the parity-specific gender gap in employment among parents from midlife onward. This stage of life coincides with the years when most women have completed their childbearing, and their childcare burden tends to be less heavy than it is at younger ages. However, it is during this period that the long-term consequences of occupational and wage gaps, as well as the loss of work experience, become particularly pronounced. In contrast, fathers, when compared to childless men, tend to enjoy wage premiums, career advantages, and greater occupational stability. Whether gender differences in employment patterns exist beyond the childbearing years will have additional effects on cumulative lifetime income and retirement income levels (Kahn et al., 2014, Muller et al., 2020), and will thus increase gender disparities in later life.

Our study also seeks to address the research gap regarding the relationship between fathers’ employment trajectories from midlife and the number of children, an area that has received relatively little attention, with only a few studies dedicated to this issue (e.g., Baranowska-Rataj & Matysiak, 2022; Sigle-Rushton et al., 2013). This complementary perspective is valuable in light of the current attitudes regarding mothers’ and fathers’ roles in society and the family, which have shifted toward favoring greater gender equality.

In this study, we illustrate the implications of parity on employment between age 40 and 74. Our analysis explores employment disparities within and between genders and across parities, going beyond the gender gap among working parents. We consider the entire population of men and women within these ages, summarizing their employment trajectories through expected years of employment by parity. This approach enables us to understand how parenthood intersects with employment trajectories from midlife onward, providing a more nuanced perspective on its consequences on both genders from midlife onward. By applying multistate life-table models to data on three different countries—Finland, Italy, and the USA—and focusing on employment expectancy as the main outcome, our study offers a demographic perspective that contributes to a better understanding of how individual characteristics, such as gender, parity, and education, intersect with the welfare state context and how their intersection affects women’s and men’s opportunities in the labor market, perpetuating gender disparities. We also assess the extent to which our approach is sensitive to the increasing age at entering parenthood and differential fertility timing. This is done through additional estimates of years in employment across birth cohorts and by narrowing our focus to older ages.

## Background and Research Questions

### Employment by Gender, Parity and Welfare Context

Past research, with some contributions dating back decades (Becker, 1981), has identified motherhood as one of the most critical factors responsible for the gender gaps in the labor market (e.g., Aisenbrey & Fasang, 2017; Killewald & Zhuo, 2019; Musick et al., 2022). The disadvantages mothers experience are likely to accumulate as the number of children grows (Baranowska-Rataj & Matysiak, 2016; Cukrowska-Torzewska & Matysiak, 2020; Doren, 2019a; Kahn et al., 2014).

While the literature investigating the relationship between fertility and women’s employment is abundant, research on the differences in mothers’ employment and earnings patterns by parity (e.g., Baranowska-Rataj & Matysiak, 2016; Doren, 2019a, 2019b; Kahn et al., 2014) is more limited, and there are relatively few comparisons of employment patterns of mothers and fathers (Baranowska-Rataj & Matysiak, 2022; Sigle-Rushton et al., 2013), and even fewer focusing on these patterns from midlife onward.

Across advanced economies, mothers are still often the main or even the only childcare providers. Women may take employment breaks during the years surrounding the birth of a child and for several years postpartum, and the consequences of such interruptions on their career prospects and earnings can last much longer (Kahn et al., 2014). In some contexts, such as in Italy, it is also common for mothers to leave the labor market altogether (Pacelli et al., 2013; Solera, 2009), to decrease their working hours, or to re-enter the labor market in a less prestigious and a less rewarding job. Taking child-related career breaks can negatively affect human capital accumulation, future employment opportunities, and desire to work. The consequences of career breaks can last until retirement, and reduce women’s financial well-being at older ages (Muller et al., 2020). Having successive births and needing to devote additional time and effort to childrearing are likely to diminish the employment opportunities of mothers (Evertsson, 2013) and may contribute to the accumulation of labor market disadvantages, especially when public policies do not support maternal employment.

Fathers, by contrast, usually take very short child-related career breaks (Bruning & Plantenga, 1999; Sigle-Rushton et al., 2013). Even in the more egalitarian Nordic countries, mothers take substantially longer leaves than fathers (NOSOSCO, 2017). More generally, there is evidence of a fatherhood advantage in terms of both wages and the probability of employment (Baranowska-Rataj & Matysiak, 2022; Glauber, 2018; Killewald, 2013; Lundberg & Rose, 2000; Mari, 2019; Weeden et al., 2016; Weinshenker, 2015; Yu & Hara, 2021). This advantage appears to be particularly large for married residential fathers compared to childless men and mothers (Killewald, 2013).

Studies attempting to explain why fathers have advantages over mothers in the labor market have focused on three main mechanisms: increased gender-specific specialization after childbirth, employers’ preferences (discrimination), and selection. It is assumed that if the mother is the primary caregiver, the father will take the role of the financial provider and will thus increase his productivity through greater work commitment. Men’s behavioral changes after entering fatherhood could increase their chances for promotion, even without increasing their work efforts (Budig & Hodges, 2010). Baranowska-Rataj and Matysiak (2022) have hypothesized that the fatherhood advantage may change the labor market position of a man with each additional child he has because of the increased financial pressure and care burden on his family. In turn, discrimination by employers is commonly experienced by mothers (Budig & England, 2001). In practice, even women of childbearing age who do not have children may face discrimination due to their potential to become a mother.

Mothers’ preferences for prioritizing family over work (Hakim, 2000) or for having a less demanding job are forms of selection, and are likely important drivers of the differences in employment rates between men and women with and without children. However, selection due to individual’s preferences likely account for only some of the differences in the employment rates. For instance, according to Eurostat (2022), in 2020, Finnish and Italian mothers’ employment rates were about 80% and 53%, respectively, while the corresponding figures for fathers were 92% and 82%. Such large differences between countries cannot be fully explained by mothers’ preferences or self-selection out of the labor market, but instead emphasize the role of the welfare state context, especially considering that both Italy and Finland are among the countries with lowest low fertility (Billari & Kohler, 2004; Hellstrand et al., 2020).

#### Employment by Gender, Parity and Welfare Context—Research Questions and Hypotheses

While occupational and wage gaps between women with and without children are observed across countries, the size of the penalties mothers face varies between different institutional and cultural contexts (Baranowska-Rataj & Matysiak, 2016; Budig et al., 2012; Cukrowska-Torzewska & Matysiak, 2020; Gangl & Ziefle, 2009; Muller et al., 2020). As gendered employment trajectories are ineluctably linked to opportunity structures and cultural contexts (Elder, 1998), the employment implications of parenthood can be mitigated or exacerbated by contextual circumstances, even after the core childbearing years.

We assess and summarize how employment trajectories vary by gender and parity in three welfare state contexts: Finland, Italy, and the USA. These countries represent different institutional and cultural settings, which, in turn, affect the opportunities and conditions for mothers’ and, to a lesser degree, fathers’ employment. Recent studies have suggested that differences across countries are more relevant than differences across cohorts in work-family life configurations (Van Winkle, 2020; Van Winkle & Fasang, 2017, 2021). Finland represents the Nordic egalitarian welfare regime model with strong public support for work-family reconciliation and the dual-earner family model (Esping-Andersen, 1990; Mills & Blossfeld, 2006). Italy is characterized as a familialistic welfare regime with limited public support for families with children (Saraceno, 2016; Saraceno & Keck, 2010), while the USA is a liberal welfare regime that provides very limited public support overall (Esping-Andersen, 1990). Unlike in Italy and the USA, family policies in Finland guarantee generous family leave combined with job protection, and support mothers’ labor market participation, for instance by providing universal access to public childcare (Esping-Andersen, 2009; Ferrarini, 2006).

Countries with public policies supportive of dual-earner families and more gender-egalitarian norms, like Finland, tend to have higher levels of female employment, particularly among mothers, than other countries (Budig et al., 2012; Jalovaara & Fasang, 2020). In countries with more traditional gender roles and limited public support for working mothers, like Italy, the division of work and care is strongly gendered (Anxo et al., 2011; Solera, 2009). In such countries, mothers are primarily responsible for childcare and for other unpaid work (Craig & Mullan, 2010), while fathers increase their working hours in line with their increased financial responsibility for the family. The USA can be viewed as a universal breadwinner welfare state with very limited government intervention through social policies (Sainsbury, 1999). This passive approach often leads mothers to increase their labor market participation soon after childbirth to minimize any income losses, and evidence suggests that the motherhood gap in employment is small at ages 40 and older (Kahn et al., 2014).

Thus, in the contexts we analyze, there are gender differences in the division of childcare responsibilities, especially among parents of young children that advantage fathers. It is reasonable to expect that such differences will become weaker at older ages when the demand for childcare lessens, but that a gender imbalance in the division of unpaid and paid work among couples may still continue. Working fathers are likely to maintain employment continuity and advance in their careers, thereby consolidating their advantages from earlier career stages. A shift in the share of paid work from fathers to mothers seems unlikely unless health problems or father’s job loss necessitates an increase in the work commitment of the mother.

Based on the differences between the three country contexts, we formulate the following hypotheses. First, we assume that where the welfare state and cultural norms are supportive of mothers’ long-term labor market attachment, as in Finland, mothers have more opportunities to invest in their careers. Thus, the employment trajectories of men and women from midlife onward are hypothesized to be relatively similar, irrespective of parity, by gender in Finland (H1). Second, we hypothesize that in Italy, there are large differences in the expected years of employment between mothers and fathers, especially at higher parities, as we expect the negative effects of motherhood to cumulate with a larger number of children (H2). Third, in the USA, we expect small gender differences by parity in employment trajectories, because mothers may need to work due to the limited institutional support for families and gender norms less traditional than in Italy (H3). Thus, in Finland and the USA, despite their distinct institutional and cultural settings, we anticipate observing gender differences in employment only at higher parities, if any exist. In contrast, for Italy, we expect that gender differences in employment accumulate with parity.

### The Intersection with Education

The increasing level of education among women is one of the most important determinants of women’s growing labor force participation (Goldin & Katz, 2018). College educated women in the USA are more likely to participate in the labor force, to be employed full-time, and to have longer working lives than less educated women. (Dudel & Myrskylä, 2017). By contrast, among the least educated, women have notably shorter working lives than men (Dudel & Myrskylä, 2017), and the gender gaps in employment and earnings increase with age (OECD, 2017). The findings of research focusing on European countries are similar, although the evidence is more heterogeneous. In the Nordic countries, the gender gap in employment is generally small, irrespective of educational level—indeed, in Finland, the gap is reversed at ages close to retirement (Leinonen et al., 2018). Meanwhile, the gap is relatively large in Italy, especially among the low educated (OECD, 2017).

There is abundant evidence that highly educated mothers are more likely to be employed than less educated mothers, which supports the opportunity cost argument, i.e., the higher cost of staying at home to care for children instead of working among women with higher levels of education (Amuedo-Dorantes & Kimmel, 2005; Kahn et al., 2014). Highly educated individuals also have a wider range of alternatives and resources (Hout, 2012) and more job options, and thus more opportunities to find a flexible, family-friendly, and well paid job (Amuedo-Dorantes & Kimmel, 2005). They also often have greater means to outsource childcare and domestic work (Gonalons-Pons, 2015). Additionally, highly educated women often hold more autonomy, which can reduce job strain and work-family conflict, and may help to mitigate the motherhood penalty (Yu & Kuo, 2017). Non-monetary aspects of employment, such as opportunities for personal growth and social rewards, may also motivate especially highly educated mothers to work for pay (Gerson, 1986).

All of these factors can make it easier for the highly educated mothers to reconcile family and work, and the importance of such factors may increase with the number of children (Baranowska-Rataj & Matysiak, 2016).

However, while having higher education may facilitate a mother’s career development by providing her with access to prestigious jobs and benefits, it may also discourage her from working because the dedication required pursuing a prestigious career may be difficult for her to maintain, especially if she has young children. Complementary evidence suggests that highly educated individuals tend to hold more demanding jobs that require putting in extra work hours or taking work home. Similarly, employers may refuse to grant highly educated women flexibility or career advancement, believing that a mother cannot fully commit to her career (Correll et al., 2007). Additionally, it is relevant to note that a highly educated woman may choose to remain at home to care for the children if her husband is also highly educated. This decision may reflect a specialization within the couple that benefits the husband’s career, a concept in line with Becker’s theory (1985).

Despite the important contribution of increased education among women in reducing the gender gap in employment, it is unclear how education intersects with gender and parity in the contemporary labor markets (Gough & Noonan, 2013; Wilde et al., 2010). For mothers, having more children could translate into having increased expenses and financial needs, more family-related responsibilities, and fewer opportunities for professional development. Though, such negative consequences can be mitigated by a more equal division of responsibilities at home, which seems to be occurring more often among highly educated couples (Sullivan et al., 2014). Today, women are often better educated than men (OECD, 2021), and fathers are increasingly involved in childcare (Hobson & Fahlén, 2009). Highly educated individuals can be considered forerunners in the transition to greater gender equality (Goldscheider et al., 2015), as they often share household responsibilities and childrearing duties more equally, including in the very low fertility countries (Sullivan et al., 2014).

#### The Intersection with Education–Research Questions and Hypotheses

We assess the role of education in shaping the differences in the employment trajectories from midlife onward of parents and childless individuals across Finland, Italy and the USA. As the expected number of years of employment reflects employment continuity and the accumulation of work experience, it is, together with education, a central element of human capital (Becker, 2009). Women who invest in education often postpone childbearing in order to establish themselves in the labor market before starting a family as the opportunity costs of having children at early career stages are high for them (Ní Bhrolcháin & Beaujouan, 2012). However, the evidence on the intersecting effects of education and parity on employment from midlife onward for both mothers and fathers is lacking. In 2015, the gender gap in the employment rate among the highly educated parents aged 25–54 with at least one child under age 15 was about 13 percentage points in Finland, compared to 18 percentage points in Italy and 19 percentage points in the USA (OECD, 2017). In these countries, the corresponding gap among the least educated was larger, at 32, 43, and 41 percentage points, respectively.

In light of the different welfare state contexts discussed in the paragraph 2.1.1 and the theoretical and contextual differences mentioned earlier, we formulate the following hypotheses regarding the role of education:

- In Finland, we expect to find small gender differences in employment among the highly educated, regardless of parity. Among the less educated Finns, we anticipate moderate gender differences, with a larger employment gap at higher parities.
- In Italy and the USA, we predict that the employment gap between mothers and fathers will be reduced among the highly educated, though to a lesser extent than in Finland. We also expect that this employment gap may persist at higher parities in these countries.
- Among the less educated, we anticipate observing a substantial gender gap in employment that increases with parity in Italy. In the USA, the size of the gender gap in employment is expected to be intermediate between that observed in Finland and Italy, with a stronger effect at higher parities.

Taken together, we hypothesize that gender differences in employment trajectories by parity will decrease with education in all contexts (H4).

## Methods and Data

### Methods

To estimate the employment trajectories and the expected number of years of employment, joblessness, and retirement from ages 40 to 74 by gender, parity, and education, we use a multistate approach (Dudel & Myrskylä, 2017; Hoem, 1977). The multistate approach extends classical event history analysis, as individuals can move back and forth between several competing states. The movement depends on the probabilities of transitioning from one state to another; e.g., the probability of moving from employment to retirement.

Individuals move between the labor force states (employment, joblessness, retirement) and eventually die. In the case of Italy and the USA, for which the data are biennial, individuals are censored at age 76. In the case of Finland, individuals are censored at age 75 as the data allow for the estimation of single-year transitions. Labor market states are “transient,” as individuals move across them. Death is an “absorbing” state, meaning that the state cannot be left. The set of all states is the “state space.” In our model, the state space covers aging by combining age 40 to age 74 with each of the transient states: e.g., “aged 68 and employed,” “aged 69 retired.” This approach allows us to capture not only the differences in the probability of moving between unemployment, employment, retirement and death, but also how these transition probabilities vary with age.

Movements across states are modeled through transition probabilities that we estimate through discrete-time event history models using multinomial logistic regressions (Allison, 1982). The state at time t + 1 is a function of the state at time t, and of additional variables; in the main specification, we control for age, education, marital status, and the number of children. Additionally, we include country-specific covariates: area of residence in Italy, race/ethnicity in the USA, and nativity in Finland. Age is modeled as a cubic restricted spline (Yee & Wild, 1996). Education is interacted with number of children to account for the potential heterogeneity in employment trajectories by education across parities. We stratify the sample by gender, implicitly interacting gender with all the variables included in the linear predictor.

We predict from the models the transition probabilities, which are the basic quantities for obtaining the expectancies. The prediction of transition probabilities for population-level estimates are obtained by setting categorical variables to their sample proportion (e.g., proportion of married, divorced, single, widowed, by parity); the same categorical indicator(s) are set equal to one for computing group-specific estimates. For Italy, we match the obtained transition probabilities with the corresponding survival probabilities of the 2008 period life tables published by the Italian National Institute of Statistics (ISTAT, 2023) using an approach based on Dudel and Myrskylä (2017). Matched transition probabilities facilitate population comparisons and reduce the impact of inaccuracies in the mortality follow-up on the overall survival. The matching algorithm does not modify the relationship between the estimated status expectancies: that is, employment, joblessness, and retirement expectancy.^1^ The transition probabilities are organized in a matrix form separately for gender, education, and parity. The obtained matrices are then transformed into the format of the fundamental matrix (Kemeny & Snell, 1983; Taylor & Karlin, 1998), from which we compute all the quantities of interests. We compute nonparametric 95% empirical bootstrap confidence intervals (Cameron & Trivedi, 2005) based on 1,000 replications of the estimation procedure. The implemented resampling procedure preserves the longitudinal structure of the data sets.

### Data

Our main data sets are representative samples of individuals aged 40 to 74 who were resident in Finland, Italy, and the USA during the 2000–2017, 1998–2016, and 1999–2019 periods, respectively.^2^ For Finland, we use a 20% random sample of the whole population permanently resident in the country, based on Statistics Finland’s register data;^3^ the measurements used in the analyses are annual. For Italy, the data come from the panel component of the Survey on Household, Income and Wealth (SHIW) carried out by the Bank of Italy (2023). The SHIW is a nationally representative survey of the Italian population conducted biennially; each wave includes about 8,000 households (20,000 individuals). For the USA, our data come from the Panel Study on Income Dynamics (PSID). The PSID is nationally representative and has been conducted annually through 1997 and biennially thereafter. The study is produced and distributed by the Survey Research Center, Institute for Social Research, (2022), University of Michigan; as of 2019, more than 9500 families were followed.

#### Outcome

The outcome is an indicator of the individual labor force status, with four mutually exclusive categories: three categories denote the transient states of being employed, joblessness (including inactive and unemployed), and retired, while the fourth category indicates the absorbing state of being alive or dead. For the Finnish data, we obtain the corresponding statuses by referring to the main activity in the last week of the year, and aggregate individuals who are unemployed, inactive, or enrolled in education into the joblessness category. For the SHIW and the PSID, we construct this variable through the corresponding self-reported labor force status at each wave. The employed category includes only individuals who are working at each of the corresponding waves. The joblessness category includes individuals who belong to one of the following self-reported categories: unemployed, inactive, student, permanently disabled, housekeeper, or others. The retired category consists only of retired individuals.

#### Covariates

In all the model specifications, we control for age, gender, marital status, the highest educational attainment, and the number of children. All the data sources provide the year of death for those individuals who died during the study period. Marital status distinguishes between married, divorced, single, and widowed individuals. Education is initially classified according to the ISCED classification and is subsequently aggregated in three broad categories: low education (in Finland, individuals with compulsory education only, i.e., lower secondary diploma; in Italy those with lower secondary education at most; in the USA those with less than high school), medium education (in Finland individuals with an upper secondary diploma; in Italy those with a high school diploma; in the USA those with high school diploma or General Equivalency Degree—GED), and high education (in Finland individuals with a qualification higher than a secondary school diploma, e.g., a bachelor’s degree; in Italy with at least a three-year university degree; in the USA those with associate degree or higher).

In the PSID, direct information on the total number of children born to an individual is provided. In the SHIW data, we obtain the number of children born to an individual by summing up the children residing in the household with the number of children who left the household. For the Finnish sample, we reconstruct the number of children each individual has based on records on the children ever registered to the person, and the marital status of each individual from the annual records on the civil status of the person in the last week of the year.

We add context-specific controls, including race/ethnicity in the USA and area of residence in Italy because such information is very relevant for correctly capturing differences in both the labor market and the fertility profiles of individuals. In the USA case, we distinguish between non-Hispanic White, non-Hispanic Black, Hispanic, and other (for simplicity, White, Black, Hispanic, and other); the other category includes individuals of all the remaining races/ethnicities. For Italy, we consider the area of residence as three macro-regions: North, Center, and South. For Finland, we control for whether individuals are or are not native-born.

## Results

### Descriptive Results

Table 1 provides summary statistics for the three analytical samples. The Finnish sample consists of more than 8.5 million person-years. The gender composition is balanced, with 53% of individuals being women, and about 60% being married. Among the highly educated, 55% are women, while the lower educational categories do not show a prevalent gender difference. In the analytical sample for Italy, there are 42,000 person-waves (the SHIW panel and the PSID are biannual). Of those, 53% are women and about 80% are married. For Italy, the gender composition of the highly educated is that opposite of that in Finland, i.e., the share of highly educated is slightly larger among men than among women. The U.S. analytical sample includes 64,000 person-waves. In the case of the USA, gender is evenly distributed, with women comprising 52% of the sample. More than 70% of the individuals are married, and women are slightly overrepresented at all educational levels apart from at the level of bachelor’s degree or higher. Compared to Finland and the USA, Italy is also characterized by a lower overall level of education, with nine out of ten individuals having at most a high school degree.

**Table 1:** Sample size by individual characteristics (in percentages)—Finland 2000–2017, Italy 1998–2016, the USA 1999–2019

*N*	Finland		Italy		USA	
	Women	Men	Women	Men	Women	Men
	4,364,377	4,190,824	22,059	19,733	34,534	29,416
*Number of children*						
0	16.1	22.1	13.4	14.3	10.8	14.2
1	18.1	16.5	19.3	19.4	14.4	14.8
2	37.0	34.4	41.1	42.5	34.5	34.2
3 +	28.8	27.0	26.2	23.7	40.4	36.7
*Education*						
Low	30.0	32.3	67.5	63.3	12.1	12.4
Medium	36.6	39.0	23.2	26.6	42.8	40.7
High	33.5	28.7	9.3	10.1	45.1	46.9
*Marital status*						
Married	57.7	61.4	75.7	84.6	65.2	81.0
Single	15.1	20.7	7.3	8.7	9.5	5.5
Divorced	18.9	15.7	5.6	4.1	19.4	11.6
Widowed	8.3	2.1	11.4	2.6	5.9	1.9
*Country of birth*						
Other country	3.3	3.4				
Finland	96.7	96.6				
*Area*						
North			43.2	43.4		
Center			20.6	20.5		
South			36.3	36.1		
*Race/Ethnicity*						
White					56.7	63.6
Black					33.8	26.0
Hispanic					6.5	7.0
Other					2.9	3.4

### Expected Years of Employment at Age 40 by Gender and Parity

The expected number of years of life spent in employment, in joblessness, and in retirement sum up to the residual life expectancy (LE) between age 40 and age 74; that is, the total number of years of life individuals expect to live from age 40 to age 74. The results summarize what the expected number of years of employment would be if the prevalent mortality and labor force conditions of the period under study were applied to a synthetic cohort of individuals aged 40 to 74. Complete tables, including results for the expected number of years of joblessness, retirement, and total life expectancy, are provided in the appendix.

**Fig. 1:**
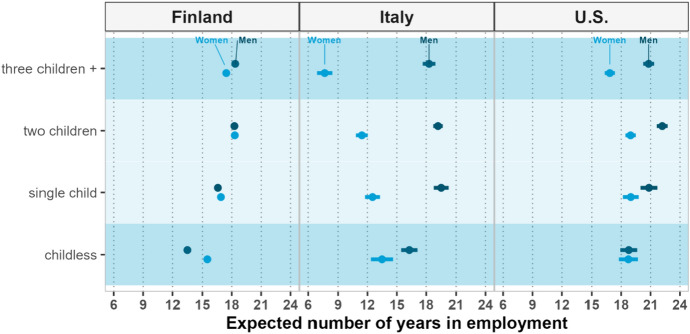
Expected years of employment at ages 40–74, by gender and parity—Finland 2000–2017, Italy 1998–2016, the USA 1999–2019. *Note*: Given the large sample size of the Finnish register data, the confidence intervals in the figure are nearly indiscernible. Complete tables with confidence interval are provided in the appendix

Figure 1 shows the expected years of employment by gender, parity, and country. For Finland, the gender differences in the expected number of years spent in employment at age 40 by parity are comparatively small. The most striking differences are observed between parities: the number of years of employment is higher among those at higher parities, up to parity two. Childless individuals have the shortest working lives among women and men. Childless women expect to work for about 15.5 years, and childless men expect to work for only about 13.5 years. This means that childless individuals expect to work less than half of their remaining life expectancy,^4^ about 47% in the case of women and 45% in the case of men. Mothers of two children and fathers of two or more children have the longest expected working life, at about 18.3 years, which corresponds to 55% of their remaining life. Mothers of three or more children expect to have a working life that is about one year shorter than that of mothers of two children, while the difference in the length of working life between fathers of two children and fathers of three or more children is negligible. The differences observed between childless individuals and parents in the Finnish context suggest that childlessness may be driven in large part by poor early labor market attachment or processes selecting individuals both out of the labor market and into remaining childless. Thus, these differences indicate that childless individuals represent a socioeconomically disadvantaged group. The very high childlessness in Table 1 supports such a perspective.

In contrast to Finland, the results for Italy show a strongly gendered relationship between the expected number of years of employment and parity. Among Italian women, the expected number of years of employment is lower among those with a larger number of children. Among Italian men, fathers are advantaged in their employment trajectories compared to childless men, but there are small differences between fathers of different parities. In stark contrast to childless women in Finland, childless women in Italy spend more years in employment (13.5 years) than mothers, and the gap between these groups is larger the more children the mother has. Mothers with three or more children are expected to work 5.9 years less than childless women. This means that at age 40, the working life of mothers with three or more children is expected to be only about 56% of the residual working life of childless women. Among men, the main difference is between fathers and childless individuals, with the latter being expected to work about 16.3 years, or around three years less than fathers. Among fathers, the expected number of remaining years of employment at age 40 is about 20, with no clear pattern according to the number of children.

The gender gap in Italy is particularly striking: for example, childless men, despite having shorter working lives than fathers, are still expected to work longer than women at any parity.

Unlike in Finland and especially Italy, in the USA, the expected length of the working life is very similar across parities zero to two for mothers, and the gender differences in expected employment emerge only at parities two and higher. What stands out in the US context is the difference in the expected years of employment between mothers of three or more children and mothers with fewer or no children. A gap of about two years in the residual working life at age 40 is an additional penalty in the labor market that mothers accumulate during their working life, given that the work interruptions increase with the number of children; it is important to note that this accumulation of disadvantage applies not only when comparing mothers to childless women, but it is also very likely when comparing mothers to fathers.

As expected, the comparison between Finland, Italy, and the USA shows differences in employment levels (Americans and Finns have a longer working life than Italians) by gender and parity are very different in the three countries, suggesting, as hypothesized, the important role of institutional and cultural factors in shaping how fertility and gender intersect in producing heterogeneity in employment trajectories.

### Expected Years in Employment at Age 40, by Gender, Education, and Parity

Education plays a prominent role in overall employment levels: the less educated individuals can expect to spend fewer years of their life in employment than their more highly educated counterparts, irrespective of their country, gender, and parity. Nevertheless, within levels of education, there are noteworthy differences across parities and gender.

**Fig. 2:**
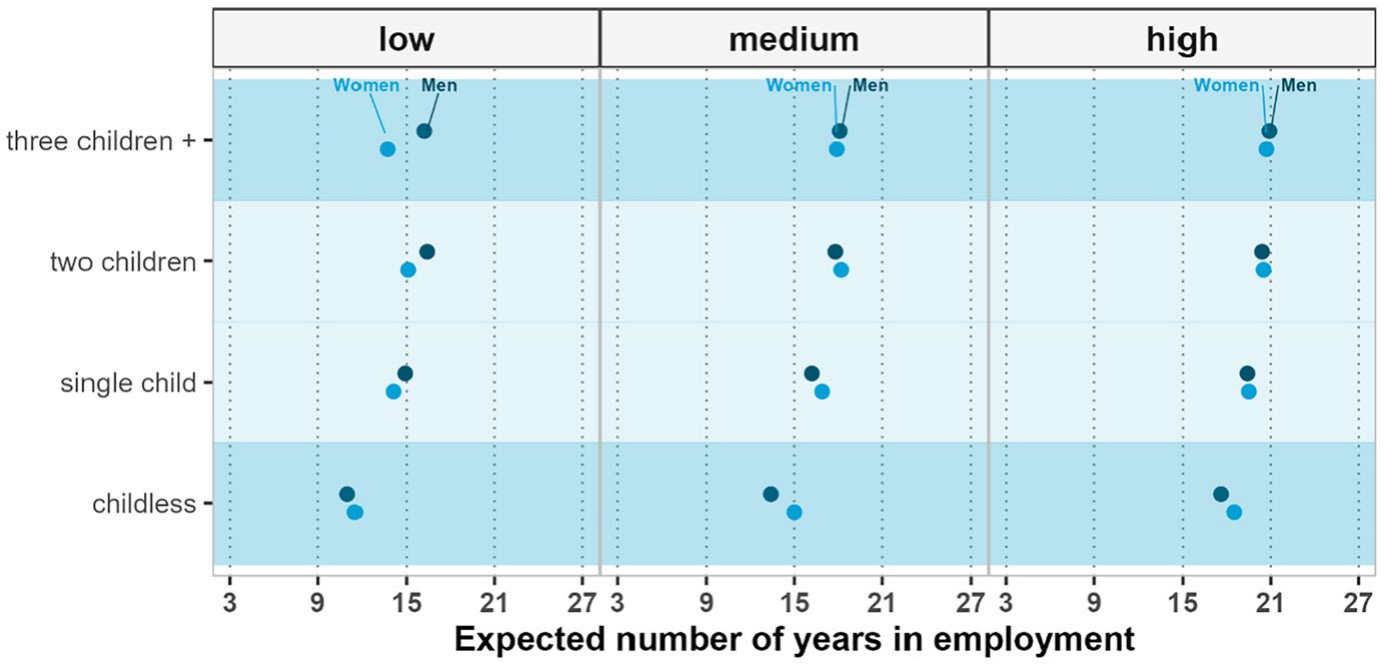
Expected years in employment at ages 40–74, by gender, parity, and education—Finland 2000–2017. *Note*: Given the large sample size of the Finnish register data, the confidence intervals in the figure are nearly indiscernible. Complete tables with confidence intervals are provided in the appendix

Figure 2 shows the results for Finland. Across educational levels, the largest difference is found between those who have less than secondary education and those who have tertiary education. In general, the results for Finland are characterized by gender similarities, although some gender gaps are observed among the less educated groups. At all educational levels, childless individuals expect to have the shortest working life, irrespective of gender. Among childless individuals, the most educated are expected to work almost twice the number of years as the least educated. Irrespective of gender, the least educated childless individuals expect at age 40 to spend fewer than 12 years in employment (less than 40% of their remaining life expectancy), while their highly educated counterparts expect to spend about 17.6 (men) or 18.5 (women) years in employment (about 55% of their remaining life expectancy).

Within levels of education, there is a degree of variation in the parity-specific employment pattern. While the expected number of years of employment increases modestly with the number of children among the highly educated, among individuals with secondary education, having two or more children is associated with having the longest working life. Mothers (fathers) of two children can expect to work about 18.2 (17.8) years at age 40 if they have a secondary school diploma, compared to around 15.1 (16.4) years if they do not have a secondary school diploma. Importantly, in the case of men, the number of children seems to play a role as important as education: the least educated fathers of two children expect to work 16.4 years, roughly the same as fathers of a single child (16.2) with a secondary school diploma.

**Fig. 3:**
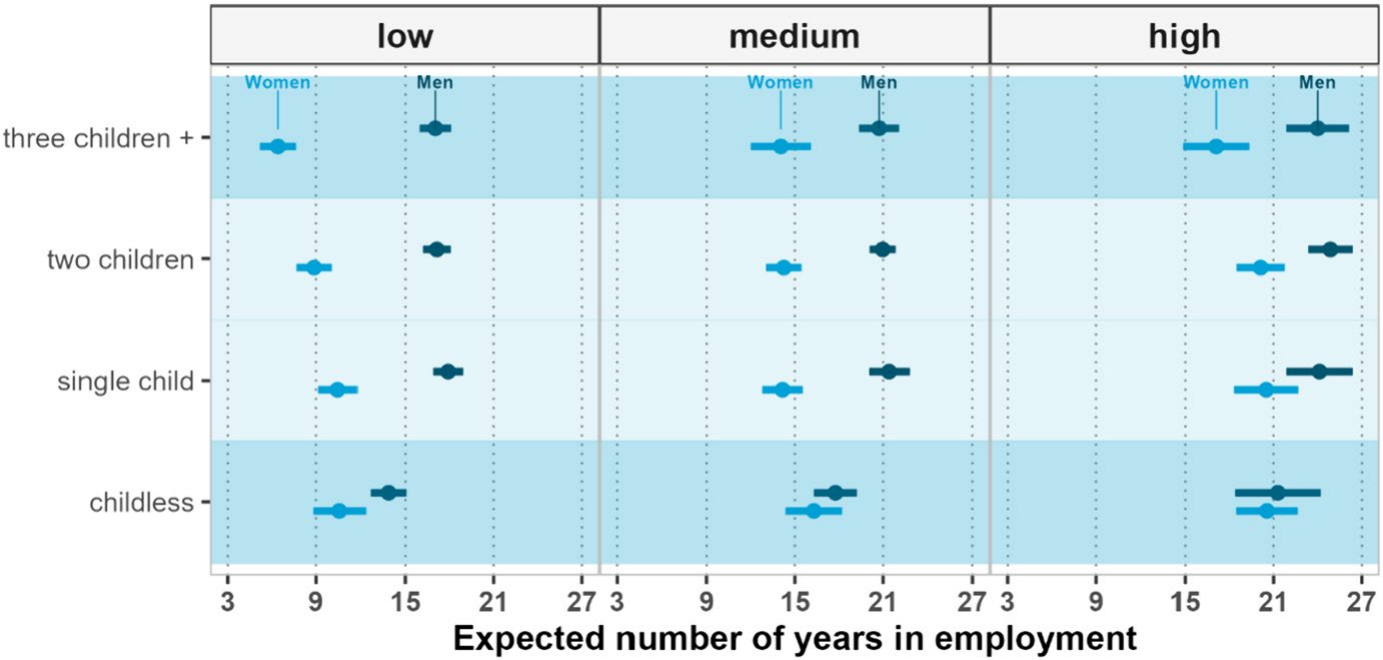
Expected years of employment at ages 40–74, by gender, parity, and education—Italy 1998–2016

Figure 3 shows the expected years of employment by gender, education, and parity in Italy. The gender differences are very large, especially among those with less than tertiary-level education. Across educational groups, fathers have a higher expected number of years of employment than childless men. Among women, those with three or more children have the shortest working life expectancy across all educational groups. The gender differences are significantly larger among the less educated groups, and the parity-specific gender differences are largest at the highest parities (3 +). The largest gap, more than 18 years, is observed when comparing highly educated fathers of two children with low educated mothers of three or more children. In other words, highly educated fathers with two children expect to work for 75% of their remaining years between ages 40 and 74. In contrast, less educated mothers with three children expect to work only 20% of their remaining life during the same age range. Across educational levels, childless men have a shorter remaining working life than other men at age 40. The gap between childless men and fathers is about three years, irrespective of education. The situation is different in the case of women, as the gap between childless and mothers increases with education; among the highly educated, childless women expect to work three years longer than mothers with three and more children. For the least educated women, the expected number of years of employment ranges from about six years among mothers with three or more children to more than ten years among childless women. Despite the strikingly low overall level of employment observed among the least educated mothers, there is a clear negative gradient by parity. Moreover, it is notable that the gender differences in employment are relatively small among the most educated men and women, and that the expected years of employment are not very different from the values observed for the Finnish and American counterparts.

In Italy, the most educated fathers expect to work about 22 to 25 years, while among the most educated women, those who are childless and those who have at most two children expect to work no less than 20 years. Mothers of three or more children expect to spend about 17 years in employment, or seven years less than their male counterparts and between three to four years less than other highly educated women.

**Fig. 4:**
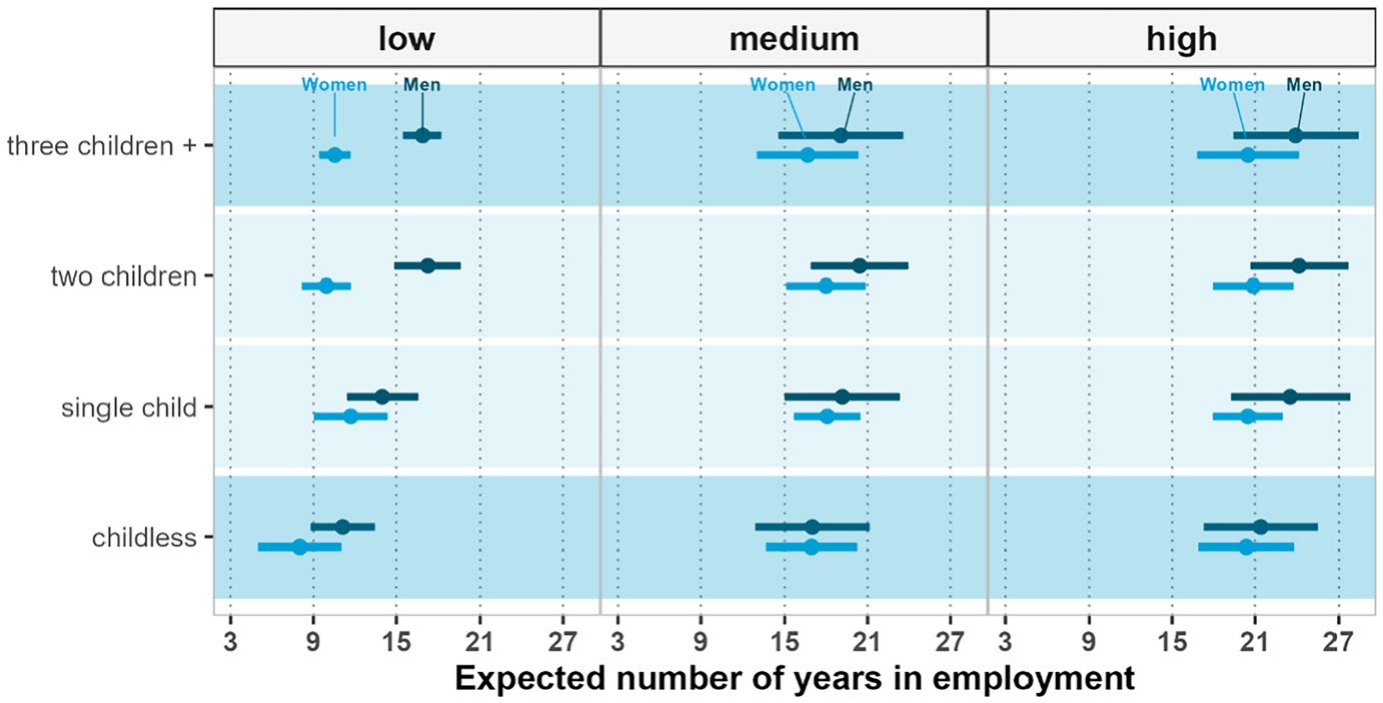
Expected years of employment at ages 40–74, by gender, parity, and education—the USA 1999–2019

Figure 4 shows the results for different educational groups in the USA. The gender differences in the number of years spent in employment at age 40 are fairly large among the least educated, while among the highly educated groups, there are moderate gender differences albeit with substantial uncertainty. Overall, parity-specific gender gaps in employment are much smaller in the USA than in Italy, but are more pronounced than in the gender-neutral case of Finland. The least educated mothers of three or more children expect to work about 10.6 years, or six years less than their male counterparts; a similar gap is found at parity two. Among the higher educated, the expected number of years of employment for mothers and for fathers at parities two and three or more increases considerably, and the gap decreases to two to four years at most. Irrespective of gender, the differences in the expected number of years of employment between childless individuals and parents are modest. Among childless individuals, the largest gender difference is found among the least educated, with women working about eight years and men working about 11 years.

Among women, the largest gap, of about 13 years, is between the least educated childless women and the most educated mothers with two children. Among men, within the positive education pattern, the expected years of employment increases with parity. Indeed, all childless individuals expect to spend fewer years working than fathers, irrespective of their number of children. More generally, within and between levels of education, we observe patterns of employment that are not as gendered as in Italy, and that are more similar to those observed for Finland. The least educated childless individuals expect to spend 40% of their remaining life expectancy in employment. In contrast, fathers with the same educational background expect to spend no less than 52% of their remaining life at work. Men with a high school degree or GED expect to work approximately 58% of their remaining life if they are childless. In comparison, the share of employment years for fathers in the same educational group is 5 to 8 points higher. Among highly educated men, the difference in the share of employment years between childless and fathers is even smaller; highly educated childless men expect to work for about 68% percent of their lives, while fathers expect to work at least 72% of their remaining life.

### Robustness Checks

The period perspective implies relatively stable age trends over time; this may seem a strong assumption given the rapidly increasing childbearing ages. We stratify our data into three cohorts to assess changes over time by considering individuals born before 1957, 1957–1966, and 1966–1977. In the three considered countries, the results show patterns very similar to those obtained on the full samples over ages 40–74 (Table 2). In the data stratified by cohorts, we estimated years of employment in the age ranges 44–74, 40–60, and 40–50, respectively.

**Table 2:** Employment expectancy by birth cohorts, gender and parity—Finland, 2000–2017—Italy, 1998–2016—the USA 1999–2019

	Number of children	Women			Men		
		Early	Mid	Late	Early	Mid	Late
Finland	Childless	12.3	14.3	7.8	10.4	12.6	7.1
	1	13.3	16.1	8.7	12.9	15.5	8.6
	2	14.3	17.7	9.4	14.1	17.2	9.4
	3 +	13.7	16.8	8.9	14.4	17.0	9.2
Italy	Childless	8.7	14.3	8.6	11.7	17.1	8.9
	1	8.4	12.6	8.2	13.5	19.8	10.8
	2	7.6	11.6	6.6	13.9	19.3	10.2
	3 +	4.8	8.6	4.7	12.9	19.3	9.9
USA	Childless	16.1	15.0	9.1	16.1	14.9	9.1
	1	16.0	14.9	9.0	17.2	16.7	10.2
	2	16.1	14.6	9.1	18.5	17.8	10.1
	3 +	13.6	13.9	7.5	17.2	16.7	9.7

As the data cover different periods, birth cohorts **Early**, **Mid** and **Late** are slightly different across Finland, Italy, and the USA. For Finland and the USA, **Early** includes cohorts born earlier than 1957 (ages 44–74); **Mid** corresponds to cohorts born between 1957 and 1966 (ages 40–60); **Late** corresponds to cohorts born between 1967–1977 (ages 40–50). For Italy, **Early** includes cohorts born earlier than 1954 (ages 44–74); **Mid** includes cohorts born between 1954 and 1964 (ages 40–60); **Late** includes cohorts born between 1965 and 1977 (ages 40–50)

Since the latest-born individuals are only observed at ages 40 to 50, the gaps in employment at higher parities, most visible for Italy and the USA, are expected to increase at ages older than 50, when re-entering the labor market is undoubtedly more complicated.

Furthermore, we analyzed samples restricted to ages 50–74.^5^ The expected number of years of employment at ages 40–50 is roughly equal to the difference between the estimates for the full age range of 40–74 and for ages 50–74. The sample restriction also reduces the potential variation across groups in the extent to which young children are present at home. In the three considered countries, the results show patterns very similar to those for ages 40–74^6^ (Table 3). Our additional analyses suggest that the employment gap observed at higher parities for mothers in Italy and the USA is unlikely to depend on older cohorts. In addition, it is improbable that small children in the household affect employment at age 50 or older.

**Table 3:** Employment expectancy and 95% CI at age 50 to age 74, by gender and parity—Finland, 2000–2017—Italy, 1998–2016—the USA 1999–2019

	Number of children	Women			Men		
		2.5%	Expectancies	97.5%	2.5%	Expectancies	97.5%
Finland	Childless	8.0	8.3	8.7	6.7	7.0	7.3
	1	8.6	8.9	9.2	8.3	8.6	8.9
	2	9.5	9.7	9.9	9.3	9.6	9.8
	3+	9.1	9.3	9.6	9.6	9.8	10.0
Italy	Childless	5.1	6.0	6.9	7.4	8.2	9.0
	1	5.0	5.6	6.1	8.5	9.2	9.9
	2	4.7	5.1	5.4	8.8	9.2	9.6
	3+	2.9	3.3	3.7	8.2	8.8	9.3
USA	Childless	10.3	11.2	12.0	10.3	11.1	11.9
	1	10.6	11.3	12.0	11.7	12.5	13.3
	2	10.7	11.2	11.7	12.7	13.2	13.8
	3+	9.4	9.8	10.3	11.8	12.3	12.8

## Discussion and Conclusions

Do mothers’ employment trajectories align with those of fathers after their childrearing burden is reduced? Does the length of working life from midlife vary by parity? This work examined the employment expectancy of individuals aged 40 to 74 in three different welfare contexts: Finland, Italy, and the USA. We compared the expected number of years of employment among women and men with different numbers of children, and provided novel evidence on the variation in employment trajectories across parities and genders in different welfare state contexts.

Altogether, as hypothesized, the study confirms that differences in employment patterns by gender and parity persist long after the core childbearing years and that education plays a greater role in mothers’ employment trajectories than in those of fathers.

Gendered differences in parity-specific employment trajectories confirm that women continue to suffer employment disparities, when compared to men, during a life stage when most women have completed fertility and their childcare burden tends to lessen. We provide further evidence that there is a large degree of variation in employment patterns by gender and by parity across countries (e.g., Baranowska-Rataj & Matysiak, 2016; Cukrowska-Torzewska & Matysiak, 2020; Gangl & Ziefle, 2009; Muller et al., 2020). Our study makes a distinct contribution by revealing that such variation is also present from midlife onward, and thus beyond the core childbearing years. The observed differences between countries underline the influence of institutional and cultural factors in shaping the employment patterns of women and men depending on their number of children. The results for Finland and the USA also illustrate that very different institutional settings may bring about striking similarities in gender gaps in overall employment levels.

More specifically, our results on Finland confirmed the hypothesis that parity-specific employment trajectories are relatively similar between genders overall (H1). We show that both childless men and women have the shortest working lives from midlife onward. These results are in line with previous findings showing that childless men and women in Finland do not have a particularly strong labor market attachment (Miettinen & Jalovaara, 2020; Nisén et al., 2018). They also complement and resonate with previous findings on the higher earnings of mothers and fathers in midlife as compared to the childless (Jalovaara & Fasang, 2020). Additional analyses (Online Appendix A) consolidate this interpretation by showing that childless individuals expect to spend more years both in joblessness and in retirement than individuals with children. In turn, we found that fathers and mothers of two or more children have the longest working lives between ages 40 and 74. Given that 60% of persons in the Finnish sample were married, we believe that these fathers and mothers follow the normative family life course of stable marriage and parenthood, as illustrated in Jalovaara and Fasang (2020). Among those with three or more children, fathers were found to work longer than mothers, which illustrates a more traditional division of work in families with multiple children even in Finland.

For Italy, as hypothesized, we found strikingly large gender differences and opposite parity-specific patterns of employment by gender (H2): among women, the expected number of years of employment decreases with parity, while among men, fathers have longer working lives from midlife onward than childless men. However, despite having shorter working lives life than fathers, childless men still expect to work longer than women, irrespective of how many children women have. These results for Italy suggest that in the analyzed cohorts the traditional separation of gender roles in the family is still dominant, as illustrated also by the high prevalence of marriage (80% of the sample are married). These results are consistent with previous research on the length of working life (Lorenti et al., 2019) and the motherhood penalty in Italy (Casarico & Lattanzio, 2021). Additional analyses (results in the supplementary material) show that childless men can expect to spend more time in joblessness and in retirement than fathers. Childless women, in turn, are expected to spend the smallest number of years in joblessness and the largest number of years in retirement. It may be assumed that individuals with a longer expectancy in both employment and retirement transitioned to retirement earlier, as the age interval considered is 40–74 for all individuals. Conversely, the number of years mothers are expected to spend in joblessness increases with parity (Online Appendix A). It is possible that some mothers with more children are not able to make sufficient pension contributions because of career breaks, and thus need to stay in the labor market longer to be able to claim a sufficient pension.

For the USA, our results confirm prior expectations by showing limited differences in the length of working life between men and women (H3). Gender differences are noticeable only between mothers and fathers of two or more children, who expect to spend the smallest number of years in employment. Overall, consistent with the previous literature on earning penalties and premiums (Kahn et al., 2014; Killewald, 2013; Killewald & Zhuo, 2019), our findings suggest that the motherhood employment penalty and the fatherhood premium at age 40 or older are relatively small, but persists for mothers and fathers who have two or more children. Moreover, mothers and fathers with three or more children expect to spend the highest number of years in joblessness and the lowest number of years in retirement (in the case of mothers), which may suggest that they have a potential need to stay in the labor market longer, but a limited ability to remain continuously employed.

Education appears to play a larger role in the employment trajectories of women than of men. As hypothesized (H4), our analyses reveal smaller gender differences in employment expectancies among the highly educated, irrespective of parity. Notably, the largest disparities in employment trajectories by parity were observed among less educated women. This advantage of higher education for women was consistent across all the countries we studied.

In Finland, education influences the duration of employment, with no interaction observed between gender and parity; this effect is consistent across all groups. These results are consistent with the literature and the consideration made for the general population of women and men. Our results for Italy show a notable interaction: as education level increases, it significantly narrows the gender gap in expected years of employment, potentially reducing it by half or more. While, in the USA, we observed an interaction between education and parity, primarily affecting the least educated individuals. Our results resonate and complement previous research, particularly with the study of Doren (2019b). Overall, these findings emphasize the critical role of education in reducing gender disparities across different labor market contexts. They suggest that education serves as a powerful means for advancing gender equality, especially in countries with higher levels of gender inequality. In such contexts, improving labor market outcomes through increased educational attainment is crucial for promoting equal opportunities for all individuals, regardless of their gender or parental status.

Our study had provided a comprehensive measure of the disadvantage (advantage) that women (men), and especially mothers (fathers), experience in the labor market from their early forties until retirement age. However, the simplicity of our measure is not without limitations. We could not evaluate how the employment trajectories of mothers and fathers unfold from the first transition to parenthood onward. Future research should explore the complete employment patterns from entry into the labor market until retirement, and evaluate the impact of different parities on the full employment trajectories. Furthermore, we could not draw causal conclusions, as we could not exclude the possibility that our estimates are affected by selection. For instance, some individuals, like mothers of three or more children, may have a stable preference for family over work that affects their fertility and their long-term employment trajectories. Indeed, we do not interpret causally the relationship between parities and observed differences in employment years.

From a broader perspective, the expected number of years of employment is a straightforward indicator that is easy to interpret, and it allowed us to compare three countries with very different welfare systems and cultures. As such, this synthetic measure of employment years has the potential to contribute to a better understanding and a more accurate identification of the groups at higher risk of labor market marginalization. Our estimates rely on biennial transitions in the case of Italy and USA, whereas Finland’s estimates are derived from annual transitions due to differences in data collection timing and frequency. Biennial transitions have fewer observed transitions compared to annual transitions, which can slightly overestimate life expectancies for Italy and the USA. Nevertheless, it is important to emphasize that the estimated patterns for gender, education, and parity remain consistent for Italy and the USA, just as they do for Finland.

We are aware that in each investigated country, large differences in employment trajectories exist across population strata; for instance, between race/ethnic groups in the USA or across macro-areas in Italy. These should be investigated in more detail in further studies. Future work can further contribute to a better understanding of the intersection between labor market and demographic processes. Indeed, new union forms are becoming increasingly relevant in many countries. Cohabitation as opposed to marriage, later marriage, and union instability may generally increase the incentives for women to participate in the labor market. However, changing family dynamics may also change the constraints for participation of both parents.

Although some of the estimated differences in the expected number of years of employment may appear small, these results should be seen as pointing to (dis)advantages that (wo)men accumulate in the labor market from age 40 onward that are additional to the (dis)advantages they accumulate up to that age. Moreover, it is worth emphasizing that while other research has mainly focused on examining employed individuals and the wage gaps due to childbearing, our estimates are based on the entire labor market experiences of the whole populations; hence, even differences that appear small can contribute significantly at the aggregate level. To put our results in perspective, it is worth recalling that over the last two decades, major reforms have been enacted, which increased the retirement age, often by a few months, to ensure the sustainability of pension systems. We argue that investing in women’s employment, and especially in mothers’ employment, should be at the top of policy-makers’ agenda, given the expected positive repercussions at the individual and the societal level, particularly for gender equality and the financial sustainability of welfare systems.

## Supplementary Information

Below is the link to the electronic supplementary material.

Supplementary file1 (DOCX 119 KB)

## Data Availability

Data from the Survey on Household Income and Wealth (SHIW) data are freely available from Bank of Italy—https://www.bancaditalia.it/. Data from the Panel Study of Income Dynamics (PSID) are freely available from the Institute for Social Research, University of Michigan—https://psidonline.isr.umich.edu/. Finnish register data are provided by Statistic Finland, but restrictions apply to the availability of these data, which were used under license for the current study, and so are not publicly available. Code for reproducibility will be made available upon request.

## Footnotes

1 Results obtained without applying the life table correction can be found in the appendix.

2 We used the most recent available data for the three countries under study. Restricting the data to a closer year range did not affect the results.

3 License nr. TK-53–1119-17.

4 The remaining life expectancy between age 40 and 74.

5 We considered a further restriction to ages 60–74, and obtained, as expected, very small residual expectancies. Thus, these results are not included, but are available upon request.

6 As employment at ages older than 74 is very rare, especially in the European context, we chose the threshold 74. Increasing the upper age limit to 80 did not result in appreciable differences in the expectancies.
