# Supplementary material for: Gendered Parenthood-Employment Gaps from Midlife: A Demographic Perspective Across Three Different Welfare Systems
Source: Eur J Popul. 2024 May 24;40(1):16. doi: 10.1007/s10680-024-09699-2 (PMC11126551; doi:10.1007/s10680-024-09699-2)
Supplement: Supplementary file 1 — Supplementary file1 (DOCX 119 KB) [file 10680_2024_9699_MOESM1_ESM.docx]

## Appendix A

Table 1 - Sample size by individual characteristics – Finland 2000-2017 (person-years)

|  |  | Women | Men | Total | Women | Men |
| --- | --- | --- | --- | --- | --- | --- |
|  |  | 4,364,377 | 4,190,824 | 8,555,201 | 51.0 | 49.0 |
| Number of children |  |  |  |  |  |  |
|  | 0 | 703,457 | 926,455 | 1,629,912 | 43.2 | 56.8 |
|  | 1 | 791,290 | 691,659 | 1,482,949 | 53.4 | 46.6 |
|  | 2 | 1,613,704 | 1,442,064 | 3,055,768 | 52.8 | 47.2 |
|  | 3+ | 1,255,926 | 1,130,646 | 2,386,572 | 52.6 | 47.4 |
| Education |  |  |  |  |  |  |
|  | Low | 1,307,219 | 1,352,250 | 2,659,469 | 49.2 | 50.8 |
|  | Medium | 1,595,206 | 1,636,241 | 3,231,447 | 49.4 | 50.6 |
|  | High | 1,461,952 | 1,202,333 | 2,664,285 | 54.9 | 45.1 |
| Marital status |  |  |  |  |  |  |
|  | Married | 2,520,410 | 2,575,019 | 5,095,429 | 49.5 | 50.5 |
|  | Single | 658,979 | 867,072 | 1,526,051 | 43.2 | 56.8 |
|  | Divorced | 823,516 | 659,720 | 1,483,236 | 55.5 | 44.5 |
|  | Widowed | 361,472 | 89,013 | 450,485 | 80.2 | 19.8 |
| Country of birth |  |  |  |  |  |  |
|  | Other country | 146,149 | 140,846 | 286,995 | 50.9 | 49.1 |
|  | Finland | 4,218,228 | 4,049,978 | 8,268,206 | 51.0 | 49.0 |

Table 2 - Sample size by individual characteristics – Italy, 1998-2016 (person-wave)

|  |  | Women | Men | Total | Women | Men |
| --- | --- | --- | --- | --- | --- | --- |
|  |  | 22,059 | 19,733 | 41,832 | 52.7 | 47.3 |
| Number of children |  |  |  |  |  |  |
|  | 0 | 2,948 | 2,826 | 5,774 | 51.1 | 48.9 |
|  | 1 | 4,259 | 3,843 | 8,102 | 52.6 | 47.4 |
|  | 2 | 9,075 | 8,413 | 17,488 | 51.9 | 48.1 |
|  | 3+ | 5,777 | 4,691 | 10,468 | 55.2 | 44.8 |
| Education |  |  |  |  |  |  |
|  | Low | 14,889 | 12,510 | 27,399 | 54.3 | 45.7 |
|  | Medium | 5,121 | 5,264 | 10,385 | 49.3 | 50.7 |
|  | High | 2,049 | 1,999 | 4,048 | 50.6 | 49.4 |
| Marital status |  |  |  |  |  |  |
|  | Married | 16,701 | 16,734 | 33,435 | 50.0 | 50.0 |
|  | Single | 1,609 | 1,717 | 3,326 | 48.4 | 51.6 |
|  | Divorced | 1,228 | 816 | 2,044 | 60.1 | 39.9 |
|  | Widowed | 2,521 | 506 | 3,027 | 83.3 | 16.7 |
| Area |  |  |  |  |  |  |
|  | North | 9,525 | 8,583 | 18,108 | 52.6 | 47.4 |
|  | Center | 4,534 | 4,059 | 8,593 | 52.8 | 47.2 |
|  | South | 8,000 | 7,131 | 15,131 | 52.9 | 47.1 |

Table 3 - Sample size by individual characteristics – the U.S. 1999-2019 (person-wave)

|  |  | Women | Men | Total | Women | Men |
| --- | --- | --- | --- | --- | --- | --- |
|  |  | 34,534 | 29,416 | 63,950 | 54.0 | 46.0 |
| Number of children |  |  |  |  |  |  |
|  | 0 | 3,716 | 4,184 | 7,900 | 47.0 | 53.0 |
|  | 1 | 4,975 | 4,366 | 9,341 | 53.3 | 46.7 |
|  | 2 | 11,902 | 10,056 | 21,958 | 54.2 | 45.8 |
|  | 3+ | 13,941 | 10,810 | 24,751 | 56.3 | 43.7 |
| Education |  |  |  |  |  |  |
|  | Low | 4,178 | 3,653 | 7,831 | 53.4 | 46.6 |
|  | Medium | 14,773 | 11,962 | 26,735 | 55.3 | 44.7 |
|  | High | 15,583 | 13,801 | 29,384 | 53.0 | 47.0 |
| Marital status |  |  |  |  |  |  |
|  | Married | 22,533 | 23,837 | 46,370 | 48.6 | 51.4 |
|  | Single | 3,269 | 1,623 | 4,892 | 66.8 | 33.2 |
|  | Divorced | 6,708 | 3,406 | 10,114 | 66.3 | 33.7 |
|  | Widowed | 2,024 | 550 | 2,574 | 78.6 | 21.4 |
| Race/Ethnicity |  |  |  |  |  |  |
|  | White | 19,598 | 18,712 | 38,310 | 51.2 | 48.8 |
|  | Black | 11,678 | 7,660 | 19,338 | 60.4 | 39.6 |
|  | Hispanic | 2,259 | 2,048 | 4,307 | 52.4 | 47.6 |
|  | other | 999 | 996 | 1,995 | 50.1 | 49.9 |

Table 4 – Women life expectancy and 95% CI at age 40 to age 74, by labor force status and number of children – Finland, 2000-17

| Status | Number of children | 2.5% | Expectancies | 97.5% |
| --- | --- | --- | --- | --- |
|  |  |  |  |  |
| Employment | childless | 15.4 | 15.5 | 15.6 |
|  | 1 | 16.9 | 16.9 | 17.0 |
|  | 2 | 18.2 | 18.3 | 18.3 |
|  | 3+ | 17.3 | 17.4 | 17.4 |
| Joblessness | childless | 4.4 | 4.5 | 4.5 |
|  | 1 | 4.0 | 4.0 | 4.1 |
|  | 2 | 2.9 | 3.0 | 3.0 |
|  | 3+ | 3.8 | 3.9 | 3.9 |
| Retirement | childless | 12.3 | 12.4 | 12.4 |
|  | 1 | 11.9 | 12.0 | 12.0 |
|  | 2 | 11.9 | 11.9 | 11.9 |
|  | 3+ | 11.9 | 11.9 | 12.0 |
| Life Expectancy | childless | 32.3 | 32.4 | 32.4 |
|  | 1 | 32.9 | 32.9 | 33.0 |
|  | 2 | 33.2 | 33.2 | 33.2 |
|  | 3+ | 33.2 | 33.2 | 33.2 |

Table 5 – Men life expectancy and 95% CI at age 40 to age 74, by labor force status and number of children – Finland, 2000-17

| Status | Number of children | 2.5% | Expectancies | 97.5% |
| --- | --- | --- | --- | --- |
|  |  |  |  |  |
| Employment | childless | 13.4 | 13.5 | 13.6 |
|  | 1 | 16.5 | 16.6 | 16.6 |
|  | 2 | 18.3 | 18.3 | 18.4 |
|  | 3+ | 18.4 | 18.4 | 18.5 |
| Joblessness | childless | 5.4 | 5.4 | 5.5 |
|  | 1 | 3.8 | 3.8 | 3.9 |
|  | 2 | 2.6 | 2.7 | 2.7 |
|  | 3+ | 2.7 | 2.8 | 2.8 |
| Retirement | childless | 11.4 | 11.4 | 11.5 |
|  | 1 | 11.1 | 11.1 | 11.2 |
|  | 2 | 11.1 | 11.1 | 11.1 |
|  | 3+ | 11.0 | 11.0 | 11.1 |
| Life Expectancy | childless | 30.1 | 30.2 | 30.2 |
|  | 1 | 31.4 | 31.5 | 31.5 |
|  | 2 | 32.1 | 32.1 | 32.1 |
|  | 3+ | 32.1 | 32.1 | 32.1 |

Table 6 – Women life expectancy and 95% CI at age 40 to age 74, by labor force status and number of children – Italy, 1998-2016

| Status | Number of children | 2.5% | Expectancies | 97.5% |
| --- | --- | --- | --- | --- |
|  |  |  |  |  |
| Employment | childless | 12.4 | 13.5 | 14.6 |
|  | 1 | 11.8 | 12.5 | 13.3 |
|  | 2 | 10.8 | 11.4 | 12.0 |
|  | 3+ | 6.8 | 7.6 | 8.4 |
| Joblessness | childless | 5.3 | 6.1 | 6.9 |
|  | 1 | 7.7 | 8.5 | 9.3 |
|  | 2 | 10.6 | 11.3 | 11.9 |
|  | 3+ | 14.9 | 15.8 | 16.7 |
| Retirement | childless | 13.5 | 14.3 | 15.1 |
|  | 1 | 12.0 | 12.7 | 13.3 |
|  | 2 | 10.2 | 10.7 | 11.1 |
|  | 3+ | 9.6 | 10.1 | 10.7 |
| Life Expectancy | childless | 33.7 | 33.8 | 34.0 |
|  | 1 | 33.6 | 33.7 | 33.8 |
|  | 2 | 33.3 | 33.4 | 33.5 |
|  | 3+ | 33.4 | 33.6 | 33.7 |

Table 7 – Men life expectancy and 95% CI at age 40 to age 74, by labor force status and number of children – Italy, 1998-2016

| Status | Number of children | 2.5% | Expectancies | 97.5% |
| --- | --- | --- | --- | --- |
|  |  |  |  |  |
| Employment | childless | 15.4 | 16.3 | 17.1 |
|  | 1 | 18.8 | 19.5 | 20.3 |
|  | 2 | 18.7 | 19.2 | 19.7 |
|  | 3+ | 17.6 | 18.3 | 18.9 |
| Joblessness | childless | 2.5 | 3.0 | 3.5 |
|  | 1 | 0.5 | 0.9 | 1.2 |
|  | 2 | 0.8 | 1.1 | 1.4 |
|  | 3+ | 1.4 | 1.8 | 2.2 |
| Retirement | childless | 11.9 | 12.9 | 13.9 |
|  | 1 | 11.3 | 12.1 | 12.9 |
|  | 2 | 11.7 | 12.2 | 12.6 |
|  | 3+ | 11.4 | 12.0 | 12.5 |
| Life Expectancy | childless | 31.3 | 32.2 | 33.1 |
|  | 1 | 31.9 | 32.5 | 33.1 |
|  | 2 | 32.2 | 32.5 | 32.7 |
|  | 3+ | 31.8 | 32.1 | 32.4 |

Table 8 – Women life expectancy and 95% CI at age 40 to age 74, by labor force status and number of children – Italy, 1998-2016 – No life table correction

| Status | Number of children | 2.5% | Expectancies | 97.5% |
| --- | --- | --- | --- | --- |
|  |  |  |  |  |
| Employment | childless | 13.1 | 14.3 | 15.4 |
|  | 1 | 12.2 | 13.0 | 13.8 |
|  | 2 | 11.2 | 11.8 | 12.4 |
|  | 3+ | 7.6 | 8.4 | 9.2 |
| Inactivity | childless | 5.0 | 5.8 | 6.7 |
|  | 1 | 7.7 | 8.5 | 9.3 |
|  | 2 | 10.5 | 11.2 | 11.8 |
|  | 3+ | 14.5 | 15.4 | 16.3 |
| Retirement | childless | 13.9 | 14.7 | 15.5 |
|  | 1 | 12.6 | 13.3 | 13.9 |
|  | 2 | 10.9 | 11.4 | 11.8 |
|  | 3+ | 10.1 | 10.7 | 11.3 |
| Life Expectancy | childless | 34.7 | 34.8 | 35.0 |
|  | 1 | 34.6 | 34.7 | 34.9 |
|  | 2 | 34.1 | 34.3 | 34.6 |
|  | 3+ | 34.3 | 34.5 | 34.7 |

Table 9 – Men life expectancy and 95% CI at age 40 to age 74, by labor force status and number of children – Italy, 1998-2016 - No life table correction

| Status | Number of children | 2.5% | Expectancies | 97.5% |
| --- | --- | --- | --- | --- |
|  |  |  |  |  |
| Employment | childless | 15.8 | 16.7 | 17.5 |
|  | 1 | 19.2 | 20.0 | 20.8 |
|  | 2 | 19.0 | 19.6 | 20.1 |
|  | 3+ | 18.1 | 18.8 | 19.4 |
| Inactivity | childless | 2.4 | 2.9 | 3.5 |
|  | 1 | 0.5 | 0.9 | 1.2 |
|  | 2 | 0.8 | 1.1 | 1.3 |
|  | 3+ | 1.4 | 1.8 | 2.2 |
| Retirement | childless | 12.8 | 14.0 | 15.2 |
|  | 1 | 12.0 | 12.9 | 13.9 |
|  | 2 | 12.6 | 13.1 | 13.6 |
|  | 3+ | 12.2 | 12.9 | 13.5 |
| Life Expectancy | childless | 32.5 | 33.6 | 34.7 |
|  | 1 | 33.0 | 33.8 | 34.7 |
|  | 2 | 33.3 | 33.7 | 34.1 |
|  | 3+ | 33.0 | 33.4 | 33.8 |

Table 10 – Women life expectancy and 95% CI at age 40 to age 74, by labor force status and number of children – the U.S., 1999-2019

| Status | Number of children | 2.5% | Expectancies | 97.5% |
| --- | --- | --- | --- | --- |
|  |  |  |  |  |
| Employment | childless | 17.8 | 18.7 | 19.7 |
|  | 1 | 18.2 | 19.0 | 19.8 |
|  | 2 | 18.4 | 19.0 | 19.5 |
|  | 3+ | 16.3 | 16.8 | 17.4 |
| Joblessness | childless | 4.6 | 5.2 | 5.8 |
|  | 1 | 5.1 | 5.6 | 6.2 |
|  | 2 | 5.7 | 6.1 | 6.4 |
|  | 3+ | 8.4 | 8.9 | 9.3 |
| Retirement | childless | 6.9 | 7.7 | 8.5 |
|  | 1 | 7.3 | 8.0 | 8.7 |
|  | 2 | 7.4 | 7.8 | 8.3 |
|  | 3+ | 6.2 | 6.6 | 6.9 |
| Life Expectancy | childless | 30.8 | 31.6 | 32.5 |
|  | 1 | 31.9 | 32.6 | 33.3 |
|  | 2 | 32.4 | 32.8 | 33.3 |
|  | 3+ | 31.9 | 32.3 | 32.7 |

Table 11 – Men life expectancy and 95% CI at age 40 to age 74, by labor force status and number of children – the U.S., 1999-2019

| Status | Number of children | 2.5% | Expectancies | 97.5% |
| --- | --- | --- | --- | --- |
|  |  |  |  |  |
| Employment | childless | 17.9 | 18.8 | 19.6 |
|  | 1 | 20.0 | 20.8 | 21.7 |
|  | 2 | 21.6 | 22.2 | 22.8 |
|  | 3+ | 20.2 | 20.8 | 21.4 |
| Joblessness | childless | 3.6 | 4.1 | 4.6 |
|  | 1 | 2.8 | 3.2 | 3.6 |
|  | 2 | 2.0 | 2.2 | 2.5 |
|  | 3+ | 3.1 | 3.3 | 3.6 |
| Retirement | childless | 6.5 | 7.3 | 8.1 |
|  | 1 | 6.0 | 6.7 | 7.5 |
|  | 2 | 7.0 | 7.4 | 7.9 |
|  | 3+ | 6.6 | 7.0 | 7.5 |
| Life Expectancy | childless | 29.2 | 30.1 | 31.1 |
|  | 1 | 30.0 | 30.8 | 31.7 |
|  | 2 | 31.4 | 31.9 | 32.4 |
|  | 3+ | 30.7 | 31.2 | 31.7 |

Table 12 – Women life expectancy and 95% CI at age 40 to age 74, by labor force status, education, and number of children – Finland, 2000-2017

| Status | Education | Number of children | 2.5% | Expectancies | 97.5% |
| --- | --- | --- | --- | --- | --- |
| Employment | Low | Childless | 11.3 | 11.5 | 11.7 |
|  |  | 1 | 13.9 | 14.1 | 14.3 |
|  |  | 2 | 15.0 | 15.1 | 15.2 |
|  |  | 3 + | 13.6 | 13.7 | 13.9 |
|  | Medium | Childless | 14.8 | 15.0 | 15.1 |
|  |  | 1 | 16.8 | 16.9 | 17.0 |
|  |  | 2 | 18.1 | 18.2 | 18.3 |
|  |  | 3 + | 17.8 | 17.9 | 18.0 |
|  | High | Childless | 18.3 | 18.5 | 18.6 |
|  |  | 1 | 19.4 | 19.5 | 19.6 |
|  |  | 2 | 20.4 | 20.5 | 20.6 |
|  |  | 3 + | 20.7 | 20.7 | 20.8 |
| Joblessness | Low | Childless | 6.6 | 6.8 | 7.0 |
|  |  | 1 | 5.8 | 5.9 | 6.1 |
|  |  | 2 | 5.1 | 5.2 | 5.3 |
|  |  | 3 + | 6.2 | 6.4 | 6.5 |
|  | Medium | Childless | 4.2 | 4.3 | 4.4 |
|  |  | 1 | 3.7 | 3.8 | 3.9 |
|  |  | 2 | 3.0 | 3.1 | 3.1 |
|  |  | 3 + | 3.5 | 3.6 | 3.7 |
|  | High | 0 | 3.0 | 3.1 | 3.2 |
|  |  | 1 | 2.4 | 2.5 | 2.6 |
|  |  | 2 | 1.7 | 1.8 | 1.8 |
|  |  | 3 + | 1.9 | 1.9 | 2.0 |
| Retirement | Low | Childless | 12.8 | 12.9 | 13.1 |
|  |  | 1 | 12.4 | 12.5 | 12.6 |
|  |  | 2 | 12.4 | 12.5 | 12.6 |
|  |  | 3 + | 12.5 | 12.6 | 12.7 |
|  | Medium | 0 | 12.9 | 13.0 | 13.1 |
|  |  | 1 | 12.2 | 12.2 | 12.3 |
|  |  | 2 | 11.9 | 11.9 | 12.0 |
|  |  | 3 + | 11.7 | 11.7 | 11.8 |
|  | High | Childless | 11.4 | 11.5 | 11.6 |
|  |  | 1 | 11.2 | 11.3 | 11.3 |
|  |  | 2 | 11.2 | 11.3 | 11.3 |
|  |  | 3 + | 11.0 | 11.0 | 11.1 |
| LE | Low | Childless | 31.2 | 31.3 | 31.4 |
|  |  | 1 | 32.4 | 32.4 | 32.5 |
|  |  | 2 | 32.8 | 32.8 | 32.8 |
|  |  | 3 + | 32.6 | 32.7 | 32.7 |
|  | Medium | Childless | 32.3 | 32.3 | 32.4 |
|  |  | 1 | 32.9 | 32.9 | 32.9 |
|  |  | 2 | 33.2 | 33.2 | 33.2 |
|  |  | 3 + | 33.2 | 33.2 | 33.3 |
|  | High | Childless | 33.1 | 33.1 | 33.1 |
|  |  | 1 | 33.2 | 33.3 | 33.3 |
|  |  | 2 | 33.6 | 33.6 | 33.7 |
|  |  | 3 + | 33.6 | 33.6 | 33.6 |

Table 13 – Men life expectancy and 95% CI at age 40 to age 74, by labor force status, education, and number of children –Finland, 2000-2017

| Status | Education | Number of children | 2.5% | Expectancies | 97.5% |
| --- | --- | --- | --- | --- | --- |
| Employment | Low | Childless | 10.9 | 11.0 | 11.2 |
|  |  | 1 | 14.8 | 14.9 | 15.1 |
|  |  | 2 | 16.3 | 16.4 | 16.5 |
|  |  | 3 + | 16.1 | 16.2 | 16.3 |
|  | Medium | Childless | 13.3 | 13.4 | 13.5 |
|  |  | 1 | 16.1 | 16.2 | 16.3 |
|  |  | 2 | 17.7 | 17.8 | 17.9 |
|  |  | 3 + | 18.0 | 18.1 | 18.2 |
|  | High | Childless | 17.5 | 17.6 | 17.8 |
|  |  | 1 | 19.2 | 19.4 | 19.5 |
|  |  | 2 | 20.3 | 20.4 | 20.4 |
|  |  | 3 + | 20.8 | 20.9 | 20.9 |
| Joblessness | Low | Childless | 6.4 | 6.5 | 6.6 |
|  |  | 1 | 4.6 | 4.7 | 4.8 |
|  |  | 2 | 3.7 | 3.8 | 3.9 |
|  |  | 3 + | 3.9 | 4.0 | 4.1 |
|  | Medium | Childless | 5.1 | 5.2 | 5.3 |
|  |  | 1 | 3.8 | 3.9 | 4.0 |
|  |  | 2 | 2.9 | 2.9 | 3.0 |
|  |  | 3 + | 2.8 | 2.9 | 2.9 |
|  | High | 0 | 3.7 | 3.8 | 3.9 |
|  |  | 1 | 2.4 | 2.5 | 2.5 |
|  |  | 2 | 1.8 | 1.8 | 1.8 |
|  |  | 3 + | 1.6 | 1.6 | 1.7 |
| Retirement | Low | Childless | 11.6 | 11.7 | 11.8 |
|  |  | 1 | 11.2 | 11.3 | 11.4 |
|  |  | 2 | 11.3 | 11.4 | 11.4 |
|  |  | 3 + | 11.1 | 11.2 | 11.3 |
|  | Medium | 0 | 11.6 | 11.6 | 11.7 |
|  |  | 1 | 11.1 | 11.2 | 11.2 |
|  |  | 2 | 11.1 | 11.2 | 11.2 |
|  |  | 3 + | 11.0 | 11.0 | 11.1 |
|  | High | Childless | 10.4 | 10.5 | 10.6 |
|  |  | 1 | 10.5 | 10.6 | 10.7 |
|  |  | 2 | 10.7 | 10.7 | 10.8 |
|  |  | 3 + | 10.4 | 10.5 | 10.6 |
| LE | Low | Childless | 29.2 | 29.2 | 29.3 |
|  |  | 1 | 31.0 | 31.0 | 31.1 |
|  |  | 2 | 31.6 | 31.6 | 31.6 |
|  |  | 3 + | 31.3 | 31.4 | 31.4 |
|  | Medium | Childless | 30.1 | 30.2 | 30.2 |
|  |  | 1 | 31.2 | 31.3 | 31.3 |
|  |  | 2 | 31.9 | 31.9 | 31.9 |
|  |  | 3 + | 32.0 | 32.0 | 32.1 |
|  | High | Childless | 31.7 | 31.8 | 31.8 |
|  |  | 1 | 32.4 | 32.5 | 32.5 |
|  |  | 2 | 32.8 | 32.8 | 32.8 |
|  |  | 3 + | 32.8 | 32.9 | 32.9 |

Table 14 – Women life expectancy and 95% CI at age 40 to age 74, by labor force status, education, and number of children –Italy, 1998-2016

| Status | Education | Number of children | 2.5% | Expectancies | 97.5% |
| --- | --- | --- | --- | --- | --- |
| Employment | Low | Childless | 8,8 | 10,6 | 12,3 |
|  |  | 1 | 9,1 | 10,5 | 11,8 |
|  |  | 2 | 7,7 | 8,9 | 10,1 |
|  |  | 3 + | 5,2 | 6,4 | 7,6 |
|  | Medium | Childless | 14,4 | 16,3 | 18,2 |
|  |  | 1 | 12,8 | 14,2 | 15,5 |
|  |  | 2 | 13,0 | 14,3 | 15,5 |
|  |  | 3 + | 12,0 | 14,1 | 16,1 |
|  | High | Childless | 18,5 | 20,6 | 22,6 |
|  |  | 1 | 18,3 | 20,5 | 22,7 |
|  |  | 2 | 18,5 | 20,1 | 21,8 |
|  |  | 3 + | 14,8 | 17,1 | 19,4 |
| Joblessness | Low | Childless | 6,8 | 8,1 | 9,5 |
|  |  | 1 | 9,0 | 10,3 | 11,5 |
|  |  | 2 | 12,7 | 14,0 | 15,3 |
|  |  | 3 + | 15,7 | 17,1 | 18,5 |
|  | Medium | Childless | 3,9 | 5,4 | 6,8 |
|  |  | 1 | 7,3 | 8,6 | 10,0 |
|  |  | 2 | 8,1 | 9,3 | 10,4 |
|  |  | 3 + | 8,7 | 10,6 | 12,6 |
|  | High | 0 | 1,1 | 2,0 | 2,9 |
|  |  | 1 | 0,8 | 2,0 | 3,1 |
|  |  | 2 | 1,4 | 2,4 | 3,5 |
|  |  | 3 + | 3,3 | 5,5 | 7,7 |
| Retirement | Low | Childless | 12,5 | 13,5 | 14,5 |
|  |  | 1 | 11,9 | 12,6 | 13,4 |
|  |  | 2 | 10,3 | 10,8 | 11,4 |
|  |  | 3 + | 9,2 | 9,9 | 10,5 |
|  | Medium | 0 | 11,0 | 12,4 | 13,8 |
|  |  | 1 | 9,8 | 11,1 | 12,4 |
|  |  | 2 | 9,3 | 10,3 | 11,2 |
|  |  | 3 + | 7,8 | 9,2 | 10,6 |
|  | High | Childless | 9,9 | 11,7 | 13,6 |
|  |  | 1 | 9,7 | 11,9 | 14,0 |
|  |  | 2 | 9,6 | 10,9 | 12,1 |
|  |  | 3 + | 9,6 | 11,7 | 13,8 |
| LE | Low | Childless | 31,6 | 32,2 | 32,8 |
|  |  | 1 | 33,1 | 33,3 | 33,6 |
|  |  | 2 | 33,5 | 33,7 | 33,9 |
|  |  | 3 + | 33,2 | 33,4 | 33,6 |
|  | Medium | Childless | 33,7 | 34,1 | 34,5 |
|  |  | 1 | 33,4 | 33,9 | 34,4 |
|  |  | 2 | 33,4 | 33,8 | 34,2 |
|  |  | 3 + | 33,4 | 33,9 | 34,3 |
|  | High | Childless | 34,1 | 34,3 | 34,5 |
|  |  | 1 | 34,1 | 34,3 | 34,5 |
|  |  | 2 | 32,7 | 33,4 | 34,1 |
|  |  | 3 + | 34,1 | 34,3 | 34,5 |

Table 15 – Men life expectancy and 95% CI at age 40 to age 74, by labor force status, education, and number of children –Italy, 1998-2016

| Status | Education | Number of children | 2.5% | Expectancies | 97.5% |
| --- | --- | --- | --- | --- | --- |
| Employment | Low | Childless | 12,6 | 13,9 | 15,1 |
|  |  | 1 | 16,9 | 17,9 | 18,9 |
|  |  | 2 | 16,2 | 17,1 | 18,1 |
|  |  | 3 + | 16,0 | 17,0 | 18,1 |
|  | Medium | Childless | 16,3 | 17,8 | 19,2 |
|  |  | 1 | 20,1 | 21,4 | 22,8 |
|  |  | 2 | 20,1 | 21,0 | 21,9 |
|  |  | 3 + | 19,4 | 20,7 | 22,1 |
|  | High | Childless | 18,4 | 21,3 | 24,2 |
|  |  | 1 | 21,9 | 24,1 | 26,4 |
|  |  | 2 | 23,3 | 24,9 | 26,4 |
|  |  | 3 + | 21,9 | 24,0 | 26,1 |
| Joblessness | Low | Childless | 3,1 | 4,0 | 4,9 |
|  |  | 1 | 0,9 | 1,2 | 1,6 |
|  |  | 2 | 1,7 | 2,1 | 2,5 |
|  |  | 3 + | 2,0 | 2,6 | 3,2 |
|  | Medium | Childless | 1,9 | 2,6 | 3,4 |
|  |  | 1 | 0,4 | 0,8 | 1,2 |
|  |  | 2 | 0,5 | 0,7 | 1,0 |
|  |  | 3 + | 0,6 | 1,2 | 1,7 |
|  | High | 0 | 0,8 | 2,0 | 3,1 |
|  |  | 1 | 0,0 | 0,2 | 0,5 |
|  |  | 2 | 0,0 | 0,1 | 0,3 |
|  |  | 3 + | 0,0 | 0,5 | 0,9 |
| Retirement | Low | Childless | 12,3 | 13,2 | 14,2 |
|  |  | 1 | 11,7 | 12,6 | 13,5 |
|  |  | 2 | 12,2 | 12,9 | 13,6 |
|  |  | 3 + | 11,6 | 12,3 | 13,1 |
|  | Medium | 0 | 11,1 | 12,5 | 13,9 |
|  |  | 1 | 9,7 | 11,1 | 12,4 |
|  |  | 2 | 10,4 | 11,3 | 12,1 |
|  |  | 3 + | 9,7 | 11,1 | 12,4 |
|  | High | Childless | 7,5 | 10,3 | 13,1 |
|  |  | 1 | 6,9 | 9,2 | 11,4 |
|  |  | 2 | 6,9 | 8,4 | 9,9 |
|  |  | 3 + | 6,4 | 8,4 | 10,4 |
| LE | Low | Childless | 30,3 | 31,1 | 32,0 |
|  |  | 1 | 31,2 | 31,7 | 32,2 |
|  |  | 2 | 31,9 | 32,1 | 32,4 |
|  |  | 3 + | 31,6 | 32,0 | 32,3 |
|  | Medium | Childless | 32,2 | 32,9 | 33,6 |
|  |  | 1 | 32,9 | 33,3 | 33,8 |
|  |  | 2 | 32,5 | 33,0 | 33,4 |
|  |  | 3 + | 32,4 | 33,0 | 33,5 |
|  | High | Childless | 33,1 | 33,6 | 34,1 |
|  |  | 1 | 33,0 | 33,5 | 34,1 |
|  |  | 2 | 33,0 | 33,4 | 33,8 |
|  |  | 3 + | 32,3 | 32,9 | 33,5 |

Table 16 - Women life expectancy and 95% CI at age 40 to age 74, by labor force status, education, and number of children – the U.S., 1999-2019

| Status | Education | Number of children | 2.5% | Expectancies | 97.5% |
| --- | --- | --- | --- | --- | --- |
| Employment | Low | Childless | 5.0 | 8.0 | 11.0 |
|  |  | 1 | 9.0 | 11.7 | 14.4 |
|  |  | 2 | 8.2 | 9.9 | 11.7 |
|  |  | 3 + | 9.4 | 10.6 | 11.7 |
|  | Medium | Childless | 13.6 | 16.9 | 20.2 |
|  |  | 1 | 15.7 | 18.1 | 20.5 |
|  |  | 2 | 15.1 | 18.0 | 20.9 |
|  |  | 3 + | 13.0 | 16.7 | 20.3 |
|  | High | Childless | 16.9 | 20.4 | 23.8 |
|  |  | 1 | 17.9 | 20.5 | 23.0 |
|  |  | 2 | 17.9 | 20.9 | 23.8 |
|  |  | 3 + | 16.8 | 20.5 | 24.2 |
| Joblessness | Low | Childless | 10.7 | 14.4 | 18.1 |
|  |  | 1 | 9.1 | 11.9 | 14.6 |
|  |  | 2 | 12.2 | 14.1 | 16.0 |
|  |  | 3 + | 13.9 | 15.1 | 16.3 |
|  | Medium | Childless | 4.0 | 6.8 | 9.6 |
|  |  | 1 | 4.4 | 6.5 | 8.7 |
|  |  | 2 | 4.6 | 6.8 | 9.0 |
|  |  | 3 + | 6.2 | 8.4 | 10.5 |
|  | High | 0 | 0.9 | 3.8 | 6.8 |
|  |  | 1 | 2.1 | 4.3 | 6.5 |
|  |  | 2 | 2.2 | 4.6 | 6.9 |
|  |  | 3 + | 4.1 | 6.2 | 8.4 |
| Retirement | Low | Childless | 1.7 | 3.9 | 6.0 |
|  |  | 1 | 5.0 | 7.2 | 9.4 |
|  |  | 2 | 4.1 | 5.4 | 6.6 |
|  |  | 3 + | 4.3 | 4.9 | 5.5 |
|  | Medium | 0 | 6.5 | 7.6 | 8.8 |
|  |  | 1 | 5.9 | 7.3 | 8.8 |
|  |  | 2 | 6.9 | 7.7 | 8.4 |
|  |  | 3 + | 6.2 | 6.9 | 7.5 |
|  | High | Childless | 6.6 | 8.0 | 9.3 |
|  |  | 1 | 7.1 | 8.5 | 9.8 |
|  |  | 2 | 7.3 | 8.1 | 8.8 |
|  |  | 3 + | 6.1 | 6.7 | 7.3 |
| LE | Low | Childless | 21.6 | 26.3 | 31.0 |
|  |  | 1 | 28.1 | 30.8 | 33.4 |
|  |  | 2 | 27.5 | 29.4 | 31.3 |
|  |  | 3 + | 29.5 | 30.6 | 31.6 |
|  | Medium | Childless | 30.0 | 31.4 | 32.7 |
|  |  | 1 | 30.5 | 31.9 | 33.4 |
|  |  | 2 | 31.4 | 32.5 | 33.5 |
|  |  | 3 + | 30.4 | 31.9 | 33.4 |
|  | High | Childless | 30.6 | 32.2 | 33.7 |
|  |  | 1 | 31.8 | 33.3 | 34.8 |
|  |  | 2 | 32.4 | 33.5 | 34.6 |
|  |  | 3 + | 31.9 | 33.4 | 35.0 |

Table 17 – Men life expectancy and 95% CI at age 40 to age 74, by labor force status, education, and number of children – the U.S., 1999-2019

| Status | Education | Number of children | 2.5% | Expectancies | 97.5% |
| --- | --- | --- | --- | --- | --- |
| Employment | Low | Childless | 8.8 | 11.1 | 13.5 |
|  |  | 1 | 11.4 | 14.0 | 16.5 |
|  |  | 2 | 14.8 | 17.2 | 19.6 |
|  |  | 3 + | 15.5 | 16.8 | 18.2 |
|  | Medium | Childless | 12.9 | 17.0 | 21.1 |
|  |  | 1 | 15.0 | 19.2 | 23.4 |
|  |  | 2 | 16.9 | 20.4 | 24.0 |
|  |  | 3 + | 14.5 | 19.1 | 23.6 |
|  | High | Childless | 17.3 | 21.4 | 25.6 |
|  |  | 1 | 19.3 | 23.5 | 27.8 |
|  |  | 2 | 20.7 | 24.2 | 27.7 |
|  |  | 3 + | 19.4 | 23.9 | 28.4 |
| Joblessness | Low | Childless | 8.8 | 11.1 | 13.4 |
|  |  | 1 | 5.8 | 7.8 | 9.8 |
|  |  | 2 | 3.8 | 5.2 | 6.6 |
|  |  | 3 + | 5.7 | 6.7 | 7.6 |
|  | Medium | Childless | 2.7 | 5.0 | 7.3 |
|  |  | 1 | 2.2 | 3.7 | 5.2 |
|  |  | 2 | 1.5 | 2.9 | 4.3 |
|  |  | 3 + | 2.0 | 3.9 | 5.7 |
|  | High | 0 | 0.2 | 2.6 | 4.9 |
|  |  | 1 | 0.6 | 2.2 | 3.7 |
|  |  | 2 | 0.1 | 1.5 | 2.8 |
|  |  | 3 + | 0.0 | 1.9 | 3.7 |
| Retirement | Low | Childless | 4.1 | 6.4 | 8.8 |
|  |  | 1 | 3.4 | 5.1 | 6.9 |
|  |  | 2 | 5.5 | 7.1 | 8.8 |
|  |  | 3 + | 5.2 | 6.1 | 7.0 |
|  | Medium | 0 | 5.9 | 7.0 | 8.1 |
|  |  | 1 | 5.8 | 7.0 | 8.2 |
|  |  | 2 | 6.7 | 7.4 | 8.1 |
|  |  | 3 + | 6.5 | 7.4 | 8.2 |
|  | High | Childless | 6.2 | 7.4 | 8.6 |
|  |  | 1 | 5.2 | 6.5 | 7.7 |
|  |  | 2 | 6.5 | 7.2 | 8.0 |
|  |  | 3 + | 5.9 | 6.8 | 7.6 |
| LE | Low | Childless | 25.6 | 28.6 | 31.7 |
|  |  | 1 | 24.0 | 26.9 | 29.8 |
|  |  | 2 | 27.4 | 29.5 | 31.7 |
|  |  | 3 + | 28.5 | 29.6 | 30.7 |
|  | Medium | Childless | 26.6 | 29.0 | 31.4 |
|  |  | 1 | 27.5 | 29.9 | 32.2 |
|  |  | 2 | 28.7 | 30.8 | 32.8 |
|  |  | 3 + | 28.1 | 30.3 | 32.5 |
|  | High | Childless | 28.9 | 31.4 | 33.9 |
|  |  | 1 | 29.7 | 32.2 | 34.7 |
|  |  | 2 | 30.8 | 32.9 | 34.9 |
|  |  | 3 + | 30.3 | 32.6 | 34.8 |

Table 18 – Women life expectancy and 95% CI at age 50 to age 74, by labor force status and number of children – Finland, 2000-17

| Status | Number of children | 2.5% | Expectancies | 97.5% |
| --- | --- | --- | --- | --- |
|  |  |  |  |  |
| Employment | childless | 8.0 | 8.3 | 8.7 |
|  | 1 | 8.6 | 8.9 | 9.2 |
|  | 2 | 9.5 | 9.7 | 9.9 |
|  | 3+ | 9.1 | 9.3 | 9.6 |
| Joblessness | childless | 2.5 | 2.8 | 3.0 |
|  | 1 | 2.4 | 2.6 | 2.8 |
|  | 2 | 1.9 | 2.0 | 2.2 |
|  | 3+ | 2.3 | 2.5 | 2.7 |
| Retirement | childless | 11.5 | 11.8 | 12.1 |
|  | 1 | 11.6 | 11.8 | 12.1 |
|  | 2 | 11.5 | 11.7 | 11.8 |
|  | 3+ | 11.5 | 11.7 | 11.9 |
| Life Expectancy | childless | 22.8 | 22.9 | 23.0 |
|  | 1 | 23.2 | 23.2 | 23.3 |
|  | 2 | 23.4 | 23.5 | 23.5 |
|  | 3+ | 23.4 | 23.5 | 23.5 |

Table 19 – Men life expectancy and 95% CI at age 50 to age 74, by labor force status and number of children – Finland, 2000-17

| Status | Number of children | 2.5% | Expectancies | 97.5% |
| --- | --- | --- | --- | --- |
|  |  |  |  |  |
| Employment | childless | 6.7 | 7.0 | 7.3 |
|  | 1 | 8.3 | 8.6 | 8.9 |
|  | 2 | 9.3 | 9.6 | 9.8 |
|  | 3+ | 9.6 | 9.8 | 10.0 |
| Joblessness | childless | 3.1 | 3.3 | 3.5 |
|  | 1 | 2.3 | 2.5 | 2.7 |
|  | 2 | 1.7 | 1.9 | 2.0 |
|  | 3+ | 1.6 | 1.8 | 1.9 |
| Retirement | childless | 10.5 | 10.8 | 11.1 |
|  | 1 | 10.8 | 11.0 | 11.3 |
|  | 2 | 10.8 | 11.0 | 11.1 |
|  | 3+ | 10.7 | 10.9 | 11.1 |
| Life Expectancy | childless | 20.9 | 21.1 | 21.2 |
|  | 1 | 22.0 | 22.1 | 22.3 |
|  | 2 | 22.4 | 22.5 | 22.6 |
|  | 3+ | 22.4 | 22.5 | 22.6 |

Table 20 – Women life expectancy and 95% CI at age 50 to 74, by labor force status and number of children – Italy, 1998-2016

| Status | Number of children | 2.5% | Expectancies | 97.5% |
| --- | --- | --- | --- | --- |
|  |  |  |  |  |
| Employment | childless | 5.1 | 6.0 | 6.9 |
|  | 1 | 5.0 | 5.6 | 6.1 |
|  | 2 | 4.7 | 5.1 | 5.4 |
|  | 3+ | 2.9 | 3.3 | 3.7 |
| Joblessness | childless | 3.0 | 3.6 | 4.2 |
|  | 1 | 4.5 | 5.2 | 5.8 |
|  | 2 | 7.1 | 7.6 | 8.1 |
|  | 3+ | 9.7 | 10.3 | 11.0 |
| Retirement | childless | 13.5 | 14.4 | 15.2 |
|  | 1 | 12.6 | 13.2 | 13.8 |
|  | 2 | 10.6 | 11.0 | 11.5 |
|  | 3+ | 9.6 | 10.2 | 10.7 |
| Life Expectancy | childless | 23.9 | 24.0 | 24.1 |
|  | 1 | 23.8 | 23.9 | 24.0 |
|  | 2 | 23.6 | 23.7 | 23.8 |
|  | 3+ | 23.7 | 23.8 | 23.9 |

Table 21 – Men life expectancy and 95% CI at age 50 to age 74, by labor force status and number of children – Italy, 1998-2016

| Status | Number of children | 2.5% | Expectancies | 97.5% |
| --- | --- | --- | --- | --- |
|  |  |  |  |  |
| Employment | childless | 7.4 | 8.2 | 9.0 |
|  | 1 | 8.5 | 9.2 | 9.9 |
|  | 2 | 8.8 | 9.2 | 9.6 |
|  | 3+ | 8.2 | 8.8 | 9.3 |
| Joblessness | childless | 1.1 | 1.5 | 1.9 |
|  | 1 | 0.4 | 0.6 | 0.9 |
|  | 2 | 0.4 | 0.7 | 0.9 |
|  | 3+ | 0.9 | 1.2 | 1.5 |
| Retirement | childless | 11.9 | 12.9 | 13.9 |
|  | 1 | 12.2 | 13.0 | 13.8 |
|  | 2 | 12.6 | 13.0 | 13.4 |
|  | 3+ | 12.0 | 12.6 | 13.1 |
| Life Expectancy | childless | 21.9 | 22.6 | 23.3 |
|  | 1 | 22.3 | 22.8 | 23.3 |
|  | 2 | 22.7 | 22.9 | 23.1 |
|  | 3+ | 22.3 | 22.6 | 22.8 |

Table 22 – Women life expectancy and 95% CI at age 50 to age 74, by labor force status and number of children – the U.S., 1999-2019

| Status | Number of children | 2.5% | Expectancies | 97.5% |
| --- | --- | --- | --- | --- |
|  |  |  |  |  |
| Employment | childless | 10.1 | 11.9 | 11.0 |
|  | 1 | 10.4 | 11.8 | 11.1 |
|  | 2 | 10.3 | 11.3 | 10.9 |
|  | 3+ | 9.1 | 9.9 | 9.6 |
| Joblessness | childless | 2.7 | 3.7 | 3.2 |
|  | 1 | 3.2 | 4.1 | 3.6 |
|  | 2 | 3.7 | 4.3 | 4.0 |
|  | 3+ | 5.5 | 6.2 | 5.8 |
| Retirement | childless | 6.2 | 7.7 | 6.9 |
|  | 1 | 6.4 | 7.7 | 7.0 |
|  | 2 | 6.5 | 7.3 | 6.8 |
|  | 3+ | 5.5 | 6.2 | 5.8 |
| Life Expectancy | childless | 20.4 | 21.7 | 21.0 |
|  | 1 | 21.2 | 22.2 | 21.7 |
|  | 2 | 21.4 | 22.0 | 21.7 |
|  | 3+ | 20.9 | 21.5 | 21.2 |

Table 23 – Men life expectancy and 95% CI at age 50 to age 74, by labor force status and number of children – the U.S., 1999-2019

| Status | Number of children | 2.5% | Expectancies | 97.5% |
| --- | --- | --- | --- | --- |
|  |  |  |  |  |
| Employment | childless | 9.9 | 11.7 | 10.9 |
|  | 1 | 10.5 | 11.9 | 11.5 |
|  | 2 | 11.0 | 11.9 | 11.6 |
|  | 3+ | 9.9 | 10.7 | 10.5 |
| Joblessness | childless | 2.8 | 3.8 | 3.2 |
|  | 1 | 3.1 | 4.0 | 3.3 |
|  | 2 | 3.2 | 3.8 | 3.5 |
|  | 3+ | 4.9 | 5.5 | 5.1 |
| Retirement | childless | 6.2 | 7.8 | 6.8 |
|  | 1 | 6.4 | 7.7 | 6.9 |
|  | 2 | 6.5 | 7.3 | 6.8 |
|  | 3+ | 5.5 | 6.2 | 5.8 |
| Life Expectancy | childless | 20.4 | 21.7 | 21.0 |
|  | 1 | 21.3 | 22.2 | 21.7 |
|  | 2 | 21.5 | 22.1 | 21.8 |
|  | 3+ | 21.1 | 21.6 | 21.4 |

## Appendix B

# MC Multistate and coefficients tables

## Markov chain multistate models

The estimates of the incidence-based multistate models are based on transition probabilities that are obtained as predicted probabilities from the multinomial logit models. The probabilities that an individual aged x and in state j will be in state i at age x + 1, that is p(i|x, j), are then arranged in transition matrices P = [pij] separately for gender and parities. The state space consists of all the pairs between states and ages, from 40 to 74. The matrices P are blocks diagonal with the only non-zero entries on the main subdiagonal. We can calculate the time spent in each state by means of the fundamental matrix (Kemeny and Snell 1983; Taylor and Karlin 1998), which is obtained as follow:

N = (I_s_ − U)^−1^

where I_s_ is an m-by-m identity matrix, and U is an m-by-m matrix (m=n-1; that is, only the transient states are included, the absorbing state dead is excluded). The superscript of -1 is used to denote the inverse. The entry of N in the 𝑗^th^ column and the 𝑖^th^ row gives the expected time spent in state i starting from state j.

In the first step, we estimate state expectancies conditioning on the initial state, at age 40. Then, we estimate weighted state expectancies by age and state. For instance, let A(x,j) be the expected number of years in the state employed at age x for individuals starting in state j. The number of expected working years can be obtained as $A\left( x \right)=\sum_{j} A(x,j)w_{j}(x)$, with $w_{j}\left( x \right)$ equal to the fraction of individuals in state j at age x. We use weights for age 40, obtained from the sample distribution of the states over the whole study period averaged across ages 30-50, by gender, and parity. For education-specific estimates, we use the corresponding distributions.

**References**

Kemeny, J. G., & Snell, J. L. (1983). *Finite Markov Chains: With a New Appendix “Generalization of a Fundamental Matrix.”* *Undergraduate Texts in Mathematics*. Springer New York.

Taylor, H. M., & Karlin, S. (1998). *An Introduction to Stochastic Modeling*. Academic Press.

## Multinomial Coefficients

Table 24 - Coefﬁcients of the multinomial logistic regression for men, Finland 2000-2017

|  | log(mu[,2]/mu[,1]) | log(mu[,3]/mu[,1]) | log(mu[,4]/mu[,1]) |
| --- | --- | --- | --- |
|  | | | |
| (Intercept) | -4.399 | -15.470 | -18.612 |
| i=Inactive | -4.399 | -2.298 | -2.680 |
| i=Retired | 1.143 | 6.738 | 5.508 |
| Age (nc spline) | - | - | - |
| Children 1 | -0.153 | -0.137 | -0.155 |
| Children 2 | -0.288 | -0.217 | -0.318 |
| Children 3 | -0.280 | -0.250 | -0.292 |
| Low | 0.176 | 0.176 | 0.280 |
| High | -0.349 | -0.492 | -0.647 |
| Other | -0.601 | 0.761 | 0.661 |
| Single | 0.461 | 0.310 | 0.853 |
| Divorced | 0.443 | 0.245 | 0.987 |
| Widow | 0.277 | 0.196 | 0.691 |
| Children 1 x Low | 0.018 | -0.059 | -0.085 |
| Children 2 x Low | 0.014 | -0.038 | -0.058 |
| Children 3 x Lox | 0.046 | 0.008 | 0.051 |
| Children 1 x High | 0.007 | 0.189 | 0.249 |
| Children 2 x High | -0.001 | 0.223 | 0.215 |
| Children 3 x High | -0.088 | 0.188 | 0.012 |

NB: ( mu[,1] = mu[,Employed], mu[,2] = mu[,Inactive], mu[,3] = mu[,Retired], mu[,4] = mu[,Dead] )

Table 25 - Coefﬁcients of the multinomial logistic regression for women, Finland 2000-2017

|  | log(mu[,2]/mu[,1]) | log(mu[,3]/mu[,1]) | log(mu[,4]/mu[,1]) |
| --- | --- | --- | --- |
|  | | | |
| (Intercept) | -4.040 | -16.595 | -19.386 |
| i=Inactive | 4.299 | 2.185 | 2.336 |
| i=Retired | 1.510 | 6.991 | 5.944 |
| Age (nc spline) | - | - | - |
| Children 1 | -0.128 | -0.211 | -0.151 |
| Children 2 | -0.311 | -0.380 | -0.486 |
| Children 3 | -0.231 | -0.409 | -0.487 |
| Low | 0.330 | 0.194 | 0.432 |
| High | -0.373 | -0.501 | -0.522 |
| Other | -0.608 | 0.775 | 0.600 |
| Single | 0.090 | 0.057 | 0.340 |
| Divorced | 0.101 | 0.011 | 0.435 |
| Widow | 0.231 | 0.024 | 0.349 |
| Children 1 x Low | -0.028 | 0.012 | -0.190 |
| Children 2 x Low | 0.048 | 0.084 | -0.042 |
| Children 3 x Lox | 0.113 | 0.188 | 0.067 |
| Children 1 x High | -0.031 | 0.166 | 0.193 |
| Children 2 x High | -0.030 | 0.280 | 0.241 |
| Children 3 x High | -0.061 | 0.179 | 0.143 |

NB: ( mu[,1] = mu[,Employed], mu[,2] = mu[,Inactive], mu[,3] = mu[,Retired], mu[,4] = mu[,Dead] )

Table 26- Coefﬁcients of the multinomial logistic regression for men, Italy 1998-2016

|  | log(mu[,2]/mu[,1]) | log(mu[,3]/mu[,1]) | log(mu[,4]/mu[,1]) |
| --- | --- | --- | --- |
|  | | | |
| (Intercept) | -6.4 | -11.2 | -18.2 |
| i=Inactive | 3.5 | 1.6 | 1.9 |
| i=Retired | 1.0 | 4.6 | 3.8 |
| Age (nc spline) | - | - | - |
| Children 0 | 0.6 | 0.1 | 0.5 |
| Children 1 | 0.2 | -0.1 | -0.5 |
| Children 3 | 0.2 | 0.0 | 0.0 |
| Low | 0.9 | 0.5 | 0.9 |
| High | -1.7 | -0.6 | -0.9 |
| South | 0.8 | -0.3 | 0.2 |
| Centre | 0.0 | 0.0 | 0.1 |
| Single | 0.5 | 0.3 | -0.5 |
| Divorced | 0.4 | -0.2 | -1.9 |
| Widow | 0.5 | 0.2 | -0.6 |
| Children 0 x Low | -0.3 | 0.0 | 0.4 |
| Children 1 x Low | -0.5 | 0.0 | 0.8 |
| Children 3 x Lox | -0.2 | 0.0 | 0.0 |
| Children 0 x High | 1.2 | 0.1 | -0.9 |
| Children 1 x High | 0.5 | 0.2 | 0.1 |
| Children 3 x High | 1.0 | 0.0 | 0.7 |

NB: ( mu[,1] = mu[,Employed], mu[,2] = mu[,Inactive], mu[,3] = mu[,Retired], mu[,4] = mu[,Dead] )

Table 27 - Coefﬁcients of the multinomial logistic regression for women, Italy 1998-2016

|  | log(mu[,2]/mu[,1]) | log(mu[,3]/mu[,1]) | log(mu[,4]/mu[,1]) |
| --- | --- | --- | --- |
|  | | | |
| (Intercept) | -6.9 | -10.5 | -19.6 |
| i=Inactive | 4.8 | 2.6 | 2.8 |
| i=Retired | 3.4 | 4.9 | 3.9 |
| Age (nc spline) | - | - | - |
| Children 0 | 0.1 | 0.1 | -0.2 |
| Children 1 | 0.2 | 0.2 | 0.0 |
| Children 3 | -0.1 | -0.3 | -0.3 |
| Low | 0.5 | 0.3 | 0.5 |
| High | -0.9 | -0.2 | 0.4 |
| South | 0.7 | 0.2 | 0.3 |
| Centre | 0.2 | 0.1 | 0.2 |
| Single | -0.9 | 0.2 | -1.0 |
| Divorced | -1.0 | -0.3 | -1.5 |
| Widow | -0.8 | 0.6 | -0.2 |
| Children 0 x Low | 0.0 | 0.2 | 2.0 |
| Children 1 x Low | -0.2 | 0.1 | 0.6 |
| Children 3 x Lox | 0.3 | 0.4 | 0.8 |
| Children 0 x High | 0.0 | -0.2 | -11.8 |
| Children 1 x High | -0.2 | -0.1 | -12.1 |
| Children 3 x High | 0.7 | 0.5 | -12.2 |

NB: ( mu[,1] = mu[,Employed], mu[,2] = mu[,Inactive], mu[,3] = mu[,Retired], mu[,4] = mu[,Dead] )

Table 28 - Coefﬁcients of the multinomial logistic regression for men, US 1999-2019

|  | log(mu[,2]/mu[,1]) | log(mu[,3]/mu[,1]) | log(mu[,4]/mu[,1]) |
| --- | --- | --- | --- |
|  | | | |
| (Intercept) | -2.49 | -11.33 | -10.87 |
| i=Inactive | 3.15 | 2.10 | 2.49 |
| i=Retired | 2.12 | 4.05 | 3.18 |
| Age (nc spline) | - | - | - |
| Children 1 | -0.28 | -0.23 | 0.26 |
| Children 2 | -0.50 | -0.01 | 0.04 |
| Children 3 | -0.34 | -0.21 | 0.04 |
| Medium | -0.65 | -0.10 | -0.16 |
| High | -1.19 | -0.24 | -0.63 |
| Black | 0.48 | 0.25 | 0.41 |
| Latinx | -0.03 | -0.43 | -0.96 |
| Other | 0.21 | -0.24 | -0.87 |
| Single | 0.67 | 0.08 | 0.51 |
| Divorced | 0.58 | 0.34 | 0.68 |
| Widowed | 0.80 | -0.09 | 0.27 |
| Children 1 x Medium | 0.09 | 0.15 | -0.37 |
| Children 2 x Medium | 0.19 | -0.07 | -0.26 |
| Children 3 x Medium | 0.20 | 0.14 | -0.22 |
| Children 1 x High | 0.24 | -0.03 | -0.50 |
| Children 2 x High | 0.21 | -0.14 | -0.48 |
| Children 3 x High | 0.19 | -0.04 | -0.43 |

NB: ( mu[,1] = mu[,Employed], mu[,2] = mu[,Inactive], mu[,3] = mu[,Retired], mu[,4] = mu[,Dead] )

Table 29 - Coefﬁcients of the multinomial logistic regression for women, US 1999-2019

|  | log(mu[,2]/mu[,1]) | log(mu[,3]/mu[,1]) | log(mu[,4]/mu[,1]) |
| --- | --- | --- | --- |
|  | | | |
| (Intercept) | -2.62 | -11.13 | -9.91 |
| i=Inactive | 3.41 | 2.21 | 3.01 |
| i=Retired | 2.58 | 4.10 | 3.44 |
| Age (nc spline) | - | - | - |
| Children 1 | -0.35 | 0.17 | -0.84 |
| Children 2 | -0.10 | 0.06 | -0.44 |
| Children 3 | -0.15 | -0.16 | -0.80 |
| Medium | -0.90 | -0.05 | -1.18 |
| High | -1.34 | -0.10 | -1.35 |
| Black | 0.18 | 0.07 | 0.23 |
| Latinx | 0.07 | -0.40 | -0.79 |
| Other | 0.24 | -0.09 | -0.07 |
| Single | 0.08 | -0.26 | 0.72 |
| Divorced | -0.14 | -0.14 | 0.59 |
| Widowed | 0.08 | 0.26 | 0.73 |
| Children 1 x Medium | 0.32 | -0.29 | 0.69 |
| Children 2 x Medium | 0.13 | -0.16 | 0.18 |
| Children 3 x Medium | 0.31 | -0.01 | 0.66 |
| Children 1 x High | 0.43 | -0.20 | 0.39 |
| Children 2 x High | 0.17 | -0.21 | -0.12 |
| Children 3 x High | 0.42 | -0.21 | 0.17 |

NB: ( mu[,1] = mu[,Employed], mu[,2] = mu[,Inactive], mu[,3] = mu[,Retired], mu[,4] = mu[,Dead] )
